# Exploration and genetic analyses of canopy leaf pigmentation changes in soybean (*Glycine max* L.): unveiling a novel phenotype

**DOI:** 10.1007/s00122-024-04693-y

**Published:** 2024-08-13

**Authors:** Hee Jin You, Hyun Jo, Ji-Min Kim, Sung-Taeg Kang, Ngoc Ha Luong, Yeong-Ho Kim, Sungwoo Lee

**Affiliations:** 1 Department of Crop Science, College of Agriculture and Life Sciences, Chungnam National University, Daejeon, 34134 South Korea; 2 Department of Applied Biosciences, College of Agriculture and Life Sciences, Kyungpook National University, Daegu, 41566 South Korea; 3 Department of Crop Science and Biotechnology, College of Bioresource Science, Dankook University, Cheonan, Chungnam 31116 South Korea

## Abstract

**Key message:**

Pigmentation changes in canopy leaves were first reported, and subsequent genetic analyses identified a major QTL associated with levels of pigmentation changes, suggesting Glyma.06G202300 as a candidate gene*.*

**Abstract:**

An unexpected reddish-purple pigmentation in upper canopy leaves was discovered during the late reproductive stages in soybean (*Glycine max* L.) genotypes. Two sensitive genotypes, ‘Uram’ and PI 96983, exhibited anomalous canopy leaf pigmentation changes (CLPC), while ‘Daepung’ did not. The objectives of this study were to: (i) characterize the physiological features of pigmented canopy leaves compared with non-pigmented leaves, (ii) evaluate phenotypic variation in a combined recombinant inbred line (RIL) population (*N *= 169 RILs) under field conditions, and (iii) genetically identify quantitative trait loci (QTL) for CLPC via joint population linkage analysis. Comparison between pigmented and normal leaves revealed different *F*_v_/*F*_m_ of photosystem II, hyperspectral reflectance, and cellular properties, suggesting the pigmentation changes occur in response to an undefined abiotic stress. A highly significant QTL was identified on chromosome 6, explaining ~ 62.8% of phenotypic variance. Based on the QTL result, Glyma.06G202300 encoding flavonoid 3′-hydroxylase (F3′H) was identified as a candidate gene. In both Uram and PI 96983, a 1-bp deletion was confirmed in the third exon of Glyma.06G202300 that results in a premature stop codon in both Uram and PI 96983 and a truncated F3′H protein lacking important domains. Additionally, gene expression analyses uncovered significant differences between pigmented and non-pigmented leaves. This is the first report of a novel symptom and an associated major QTL. These results will provide soybean geneticists and breeders with valuable knowledge regarding physiological changes that may affect soybean production. Further studies are required to elucidate the causal environmental stress and the underlying molecular mechanisms.

**Supplementary Information:**

The online version contains supplementary material available at 10.1007/s00122-024-04693-y.

## Introduction

Soybeans [*Glycine max* (L.) Merr.] are an economically valuable leguminous seed crop. They are a major source of dietary protein and oil for millions of people, used as feed for livestock, and employed for the industrial manufacturing of thousands of goods (Nwokolo 1996). However, climate change and ever-increasing global warming have increased abiotic stress on organisms, driving the evolution of biochemical activities to manage this environmental stress (Ramakrishna and Ravishankar 2011). Plants biosynthesize hundreds of compounds that maintain internal homeostasis and improve their tolerance or resistance to diverse abiotic stresses (Haak et al. 2017; Shah and Smith 2020).

Under natural conditions, plants are constantly challenged by environmental stressors, such as heat, chilling, and drought, during their vegetative and reproductive growth stages. Plants respond by regulating their physiological, metabolic, and developmental processes (Sharma et al. 2022). Heat stress leads to the disruption of thylakoid membranes, which subsequently inhibits membrane-associated electron carriers, thereby decreasing their photosynthetic rate (Zhao et al. 2020). In soybean, the leaf photosynthetic rate and stomatal conductance were significantly decreased under extreme heat conditions (38/28 °C, day/night) compared with those under normal conditions (28/18 °C, day/night) (Djanaguiraman et al. 2011). In addition, heat stress negatively affected the accumulation of lipoxygenase, the *β*-conglycinin *β*-subunit, sucrose binding protein, and Bowman–Birk protease inhibitor in soybean seeds and disrupted the vacuole structure and membrane integrity in cells that accumulate seed storage proteins (Krishnan et al. 2020). Chilling stress usually results in several physiological responses in plants, including membrane rigidification and the accumulation of reactive oxygen species (Ruelland et al. 2009). The expression of genes involved in photosynthesis and the photosystem was dynamically changed in *Arabidopsis thaliana* when exposed to different chilling conditions (4 °C) (Liu et al. 2022). Under severe drought conditions, stressed *Maclura pomifera* plants reprogrammed themselves to reduce oxidative injury, accumulating more stable and protective osmolyte proline and activating more antioxidant enzymes that control plants (Khaleghi et al. 2019). These abiotic stresses can limit the growth and development of plants, which, in crops, results in yield losses (Lippmann et al. 2019). Therefore, a fundamental understanding of how plants perceive stress signals and respond to adverse environmental conditions is essential for continuous growth.

Pigments in leaves or fruits, such as chlorophylls, carotenoids, and anthocyanins, are key indicators of plant health and are associated with physiologically important functions in plants. Several studies have reported that chlorophyll and carotenoid (orange to yellow pigments) contents decrease when plant leaves are stressed (Gitelson and Merzlyak 1994; Merzlyak et al. 1999). Anthocyanins (purple and red pigments) may also protect leaves from environmental stresses (e.g., pH, UV radiation, and high- or low-temperature stresses) and have been extensively studied in maize (*Zea mays* L.), rice (*Oryza sativa* L.), *Arabidopsis*, and other plants (Carey et al. 2004; Chandler et al. 1989; Cone et al. 1986; Holton and Cornish 1995; Lepiniec et al. 2006; Reddy 1998). Anthocyanin concentrations are high in young leaves with low photosynthetic rates but low in mature or senescent leaves (Gamon and Surfus 1999).

Conventional assay methods for measuring phenotypic variation in crop species have some limitations, such as being labor-intensive, time-consuming, possibly inaccurate because of personal bias, or any combination of these (Dhondt et al. 2013; Tao et al. 2022). However, over the last decade, plant phenome research has made impressive progress through the application of sensors, imaging, and analytical techniques. Imaging technology, including visible light, hyperspectral, and fluorescence cameras, has become an effective tool for phenotyping. Especially, when used with unmanned aerial and ground vehicles, imaging technology can obtain highly accurate, specific phenotypic data in an easy, quick, automated, and non-destructive manner (Lowe et al. 2017; Tao et al. 2022; Xavier et al. 2017). Image-based phenotyping methods have been extensively used to assess morphological phenotypes and responses to both abiotic and biotic stresses in crops, including herbicide injury, nematodes, and bacterial and fungal pathogens (Duddu et al. 2019; O’Callaghan et al. 2018; Singh et al. 2021; Song et al. 2022; Zhou et al. 2022). A hyperspectral camera can collect spectral absorption and reflectance values at various wavebands from imaged data, and vegetation indices calculated from measured reflectance are widely used to easily obtain accurate and precise phenotypic measurements (e.g., pigmentation) in the field (Blackburn 1998; Chappelle et al. 1992; Gamon and Surfus 1999; Gitelson and Merzlyak 1994). Accurate phenotypic measurements based on hyperspectral reflectance combined with high-throughput genotyping facilitate the characterization of the genetic architecture of complex traits through genome-wide association studies and linkage mapping (Bhat et al. 2016; Wang et al. 2021; Xiao et al. 2022).

A novel phenomenon has been observed in certain soybean genotypes: the outer layer of the canopy exhibits reddish-purple pigmentation during the later reproductive stages. This phenotype has been observed in at least four years in multiple geographical locations in South Korea. The present study first reports the new physiological symptom, designated as canopy leaf pigmentation changes (CLPC). Secondly, we further characterized the physiological changes in the pigmented leaves using multiple approaches, comparing them with the green leaves of the inner canopy. Finally, genetic mapping of a quantitative trait locus (QTL) associated with CLPC was performed using joint population QTL analyses on 169 recombinant inbred lines (RILs).

## Materials and methods

### Plant materials

The Korean soybean nested association mapping (NAM) population, comprised of 27 bi-parental families with a common female parent, was developed and is available for genetic studies (Kim 2018). Of the NAM population, two RIL populations derived from the crosses of ‘Daepung’ × ‘Uram’ (NAM10) and Daepung × PI 96983 (NAM12) were used to characterize CLPC. Daepung is a high-yield Korean cultivar with resistance to bacterial pustules and tolerance to lodging (Park et al. 2005), Uram is a large-seeded, high-yield cultivar used for soybean paste with mechanical harvesting fitness (Ko et al. 2016), and PI 96983 is a Korean landrace with resistance to the soybean mosaic virus (Kiihl and Hartwig 1979; Yang et al. 2013). In brief, 20 and 16 F_1_ seeds were harvested from the first crosses (NAM10 and NAM12, respectively) in the fall of 2012 and subsequently planted to obtain seeds of 148 and 212 lines of F_2_ families in the winter of 2012. Single-seed descents were used to develop 115 and 144 RILs, respectively, of the two F_6_ populations by 2015. In 2018, F_6_-derived RIL seeds of the populations were obtained and used for the visual phenotypic evaluation of CLPC in the field in the following years. In addition, selected RILs from the two populations were further investigated via multiple analytic approaches described below. The selected RILs for each assay are listed in Table S1.

### Evaluation of levels of canopy leaf pigmentation changes in two bi-parental RIL populations

**Fig. 1:**
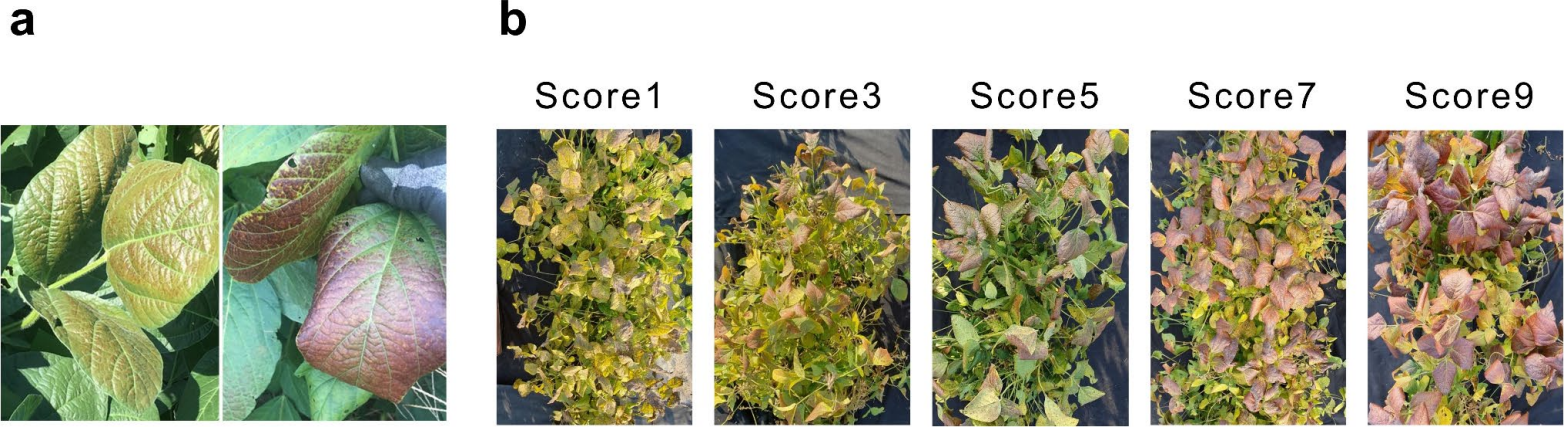
Pigmentation changes in canopy leaves of soybean plants. **a** Images showing the novel canopy leaf pigmentation changes in soybean genotypes observed in mid-August (left) and mid-September (right) in 2019. **b** The phenotypic scores characterizing the levels of the pigmentation changes based on plants in a row plot. Scores 1 and 9 indicate no and high anomalous pigmentation, respectively

The phenotypes of the two RIL populations and their parental lines were evaluated in soybean fields located in Cheonan, South Korea (36.724653, 127.260206) in 2018 and at the university farm of Chungnam National University, Daejeon, South Korea (36.3679020, 127.353206) from 2019 to 2021. Each population was treated as a subgroup for field experiments. The RILs were randomly assigned to row plots within the population with a single replication in each year: ten seeds of each genotype were sown in a 1.5 m single row with a row spacing of 1 m. The specific locations of planting varied by year within the university farm. Purple pigmentation levels in the entire plot of each genotype were visually investigated each year at the R7 stage. Leaf pigmentation levels were scored for the individual plots as 1, 3, 5, 7, and 9, representing 0–20%, 20–40%, 40–60%, 60–80%, and 80–100%, respectively. A score of 1 indicates no pigmentation change or only slight purple pigmentation in trivial proportions of canopy leaves, whereas a score of 9 indicates that pigmentation occurred uniformly in most canopy leaves (Fig. 1).

### Measurement of the maximum quantum yield of photosystem II

Based on the 2018–2020 phenotypic results, five RILs (10-013, 10-005, 10-014, 10-055, and 10-076) representing phenotypic scores 1, 3, 5, 7, and 9, respectively, were preselected from the NAM10 population. In the field test of 2021, these five RILs were purposely planted in adjacent row plots, side by side, to minimize within-field variation when comparing photosynthesis efficiency among them. A single canopy and a single middle leaf were carefully marked in two individual plants of each selected RIL. Chlorophyll fluorescence parameters from these designated leaves were measured between 3 and 5 pm. on sunny days, once a week over two months using a chlorophyll fluorometer (Junior-PAM®, Heinz Walz GmbH, Effeltrich, Germany). Two magnetic clips per leaf were attached to a part of the lamina without overlapping the vein and midrib and left for 30 min to allow dark adaptation. Then, the fluorescence intensities were measured. The maximal quantum yield (*F*_v_/*F*_m_) of photosystem (PSII) was calculated using the following equation (Kitajima and Butler 1975).

*F*_v_/*F*_m_ = (*F*_m_–*F*_o_)/*F*_m_,

where *F*_o_ is the basic fluorescence yield (relative units) recorded with low light intensities and *F*_m_ is the maximum chlorophyll fluorescence yield when the PSII reaction centers are closed by a strong light pulse (relative units).

### Measurement of hyperspectral reflectance

For each population, three insensitive (i.e., Daepung-type; score 1) and three sensitive (i.e., Uram- or PI 96983-type; score 9) RILs were preselected to further characterize the pigmentation by measuring the hyperspectral reflectance of leaves under field conditions in 2021. For NAM10, insensitive RILs included 10-012, 10-025, and 10-097 and sensitive RILs included 10-023, 10-029, and 10-102. For NAM12, insensitive RILs included 12-070, 12-071, and 12-081 and sensitive RILs did 12-031, 12-043, and 12-048. The canopy leaves of the selected RILs were marked for repeated measurements.

Hyperspectral imaging techniques were used to investigate the physiological conditions of the canopy leaves, and visible changes in the leaves were observed naturally under field conditions. Hyperspectral images were obtained from the selected RILs using Specim IQ (Specim, Oulu, Finland). Imaging was performed weekly, from 3 to 5 pm., and then, regions of interest (ROI) for each image were manually selected on symptomatic regions. The reflectance data at the spectral wavelengths of 397–1000 nm were extracted from the selected ROI of each image and analyzed to identify the range of bands that showed significantly different reflectance between the canopy leaves of insensitive and sensitive RILs using ENVI v.5.5.3 software (L3 Harris Geospatial, Boulder, CO, USA).

### Transmission electron microscopy of cell organelle ultrastructure

The canopy and middle leaves of two sensitive genotypes (10-064 and 12-002) were imaged using transmission electron microscopy (TEM) to investigate cellular changes in their pigmented canopy leaves. This was performed at the Electron Microscopy & Histology Core Facility, BioMedical Research Center, Korea Advanced Institute of Science and Technology, Daejeon, South Korea. From digitalized cell images, the thickness of cell walls was gauged, and areas of chloroplasts and starch grains were measured using ImageJ v.1.42q (available at https://imagej.nih.gov/ij/) to calculate the starch grain cover index (i.e., the proportion of the chloroplast area occupied by starch grains). Subsequently, *t*-tests were used to assess the statistical significance of differences between the canopy and middle leaves of individuals.

### SNP genotyping, genetic map construction, and QTL analyses

The parents and all RILs of the two mapping populations were genotyped using a 180 K Axiom® single-nucleotide polymorphism (SNP) array (ThermoFisher Scientific, Seoul, South Korea) (Lee et al. 2015), obtaining genotypic data for 160,028 SNPs. Raw SNP data were filtered by excluding (i) SNPs with no polymorphisms, (ii) SNPs that were undefined or with heterozygosity in either parent, (iii) SNPs with missing data for > 10% of the RILs, (iv) RILs with missing data for > 10% of SNPs, and (v) SNPs with high levels of segregation distortion (*P* < 0.001). After filtering, 20,826 SNPs for 85 RILs of the NAM10 population and 26,279 SNPs for 84 RILs of the NAM12 population were retained in the datasets and subsequently utilized for further analysis.

Genetic bin maps of the two populations were constructed using IciMapping v4.2 (Meng et al. 2015). Genotypic, phenotypic, and genetic map data were combined to identify the QTL regions. QTL analyses were conducted using the inclusive composite interval mapping method (Li et al. 2007). The logarithm of the odds (LOD) threshold was determined using a 1000-permutation test at a 5% significance level (Churchill and Doerge 1994), and the phenotypic variance (%) explained (PVE) by each QTL was calculated (Li et al. 2011). The results of the QTL analyses were depicted graphically using MapChart 2.32 (Voorrips 2002).

Based on the single-population QTL analyses of the two RIL populations, we constructed a composite map of chromosome 6 for joint population QTL analysis. The SNP alleles from Daepung were designated as the “AA” genotype, and the Uram and PI 96983 alleles were designated as the “BB” genotype. Missing and heterozygous SNPs were imputed using QTL IciMapping v4.2. The joint population QTL analysis was performed using the joint inclusive composite interval mapping method, and the PVE for each QTL was calculated (Li et al. 2011). The LOD threshold was determined based on a 1000-permutation test (Churchill and Doerge 1994).

### Isolation of RNA and quantitative reverse transcription (qRT)-PCR

Gene expression levels of Glyma.06G202300 were compared between pigmented canopy leaves and non-pigmented middle leaves within individuals. Leaves were sampled from Daepung and four sensitive genotypes from the two populations (10-023, 10-076, 12-056, and 12-063), including three biological replicates per leaf position per genotype. Leaf tissue was ground with a mortar and pestle in liquid nitrogen, and total RNA was isolated using the RNeasy Plant Mini Kit (Qiagen, Germantown, MD, USA) following the manufacturer’s instructions. Then, cDNA was transcribed using High-Capacity cDNA Reverse Transcription Kit (Thermo Fisher Scientific, Walthamm, MA, USA) according to the manufacturer’s instructions. Primers were designed to target a part of the 2nd exon of Glyma.06G202300 using Primer 3 (Untergasser et al., 2012): forward primer 5′-CGAGGGCAATGATTGGACGAA-3′ and reverse primer 5′- ACTCCAGCCAACACCATCAC-3′. The *ACT11* (Glyma.18g290800) gene (Gao et al., 2017; Jian et al., 2008) was used as the endogenous control, targeted by the forward primer 5′-CTTACATTGCCCTTGACTACGAG-3′ and reverse primer 5′-CAGAACCTCTGGACATCTGAAAC-3′. Real-time quantitative PCRs were performed using SYBR Green Q-PCR Master Mix with Low Rox (SmartGene, Daejeon, Korea), and levels of SYBR green fluorescence were detected using an Applied Biosystems™ QuantStudio™ 3 system (Thermo Fisher Scientific, Waltham, MA, USA). Each qRT-PCR reaction was performed in a 20 μL volume including 10 μL of the qPCR master mix, 0.8 μL of each primer, 2 μL of template cDNA, and 6.4 μL of dH_2_O. Amplification was conducted according to the manufacturer’s suggested thermocycler protocol, 95 °C for 2 min and then 40 cycles of 95 °C for 5 s and 62 °C for 30 s, and followed by melt curve analysis. Relative expression levels and fold changes were calculated using the comparative CT method (Schmittgen and Livak, 2008). Differential expression was compared in two ways: the expression of pigmented (canopy) leaves relative to non-pigmented (middle) leaves was calculated within individuals and compared between individuals. The expression levels of the sensitive genotypes relative to that of Daepung were calculated for pigmented canopy and non-pigmented middle leaves and compared between the two positions.

### Quantification of anthocyanins using liquid chromatography

Canopy leaves from high-scored genotypes (10-076, 10-082, 12-031, and 12-063), along with three parental lines (Daepung, Uram, and PI 96983), were utilized to analyze anthocyanin profiles. As detailed in the previous study, anthocyanin quantification was conducted using ultrahigh-performance liquid chromatography (Kim et al. 2020).

## Results

### Phenotypic variation in canopy leaf pigmentation changes

**Fig. 2:**
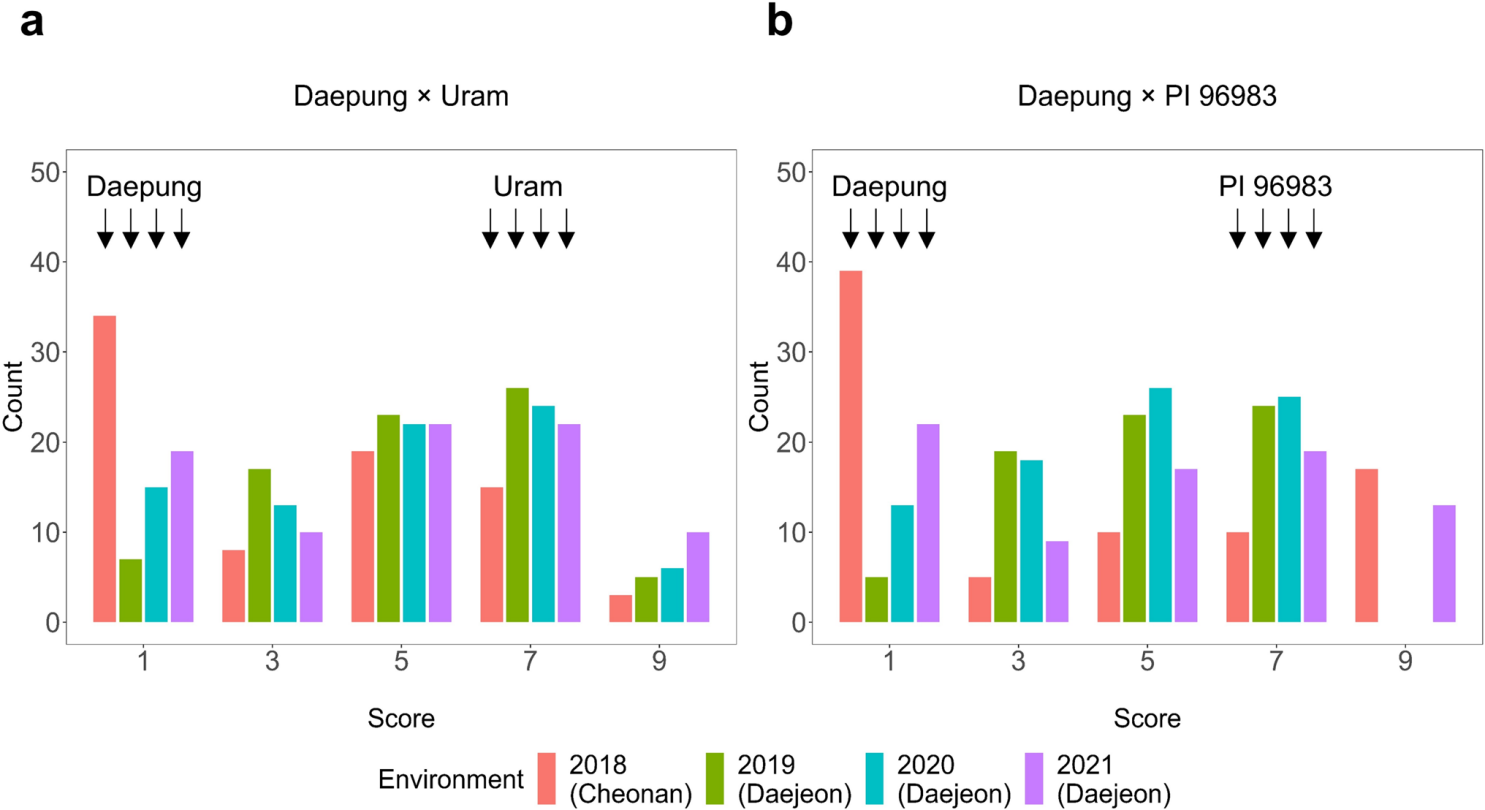
Frequency distribution of phenotypic scores for canopy leaf pigmentation changes in the Daepung × Uram (NAM 10, *N* = 85) (**a**) and Daepung × PI 96983 (NAM 12, *N* = 84) (**b**) populations

In sensitive genotypes, the pigmentation was only visible on the adaxial leaf surface of upper canopy of each row plot; leaf blade turned to reddish purple (Fig. 1). Both Uram (Score 7, sensitive) and PI 96983 (Score 7, sensitive) exhibited distinct pigmentation in their canopy leaves during the reproductive stages from mid-to-end of August at the earliest, but generally beginning from mid-to-end of September, whereas Daepung (Score 1, insensitive) had green leaves until senescence. The variation in pigmentation of canopy leaves ranged from 1 to 9, with the average of 4.6 in the combined population (Table 1; Fig. 2). There was a statistically significant difference among RILs and years (Table 2). Phenotypic distributions statistically differed by year; the means of the two populations were significantly lower in 2018 due to higher frequencies of score 1, indicating that CLPC was affected by environmental factors (Table 2). The calculated broad-sense heritability for CLPC ranged from 0.93 to 0.96 (Table 1; Table S2).

**Table 1:** Phenotypes of the parents and descriptive statistics of the phenotype scores of and recombinant inbred lines (RILs)

Population	Parents			RILs				
	Daepung	Uram	PI 96983	*N*^a^	Mean	Range	SD^b^	*H*^*2*^^c^
Daepung × Uram	1	7	–	85	4.6	1–9	2.6	0.96
Daepung × PI 96983	1	–	7	84	4.5	1–9	2.6	0.93
Combined	1	7	7	169	4.6	1–9	2.6	0.94

^a^Number of RILs assayed

^b^Standard deviation

^c^Broad-sense heritability

**Table 2:** Analysis of variance (ANOVA) tables for pigmentation in canopy leaf pigmentation changes

Source of variation	Degree of freedom	Sum of squares	Mean squares	*F*	*P*
Daepung × Uram (NAM10)					
Environment	3	109.0	36.3	38.6	< 0.0001
Genotype	84	1746.8	20.8	22.1	< 0.0001
Residual	232	218.5	0.9	–	–
Daepung × PI 96983 (NAM12)					
Environment	3	33.1	11.0	6.5	< 0.0001
Genotype	81	1770.9	21.9	12.8	< 0.0001
Residual	229	391.8	1.7	–	–
Combined					
Environment	3	129	43.04	31.973	< 0.0001
Population	1	0.29	0.29	0.214	0.644
Genotype	165	3517	21.31	15.834	< 0.0001
Residual	464	625	1.35	–	–

### Decreased maximum quantum yield (***F***_v_/***F***_m_) of PSII in pigmented canopy leaves

**Fig. 3:**
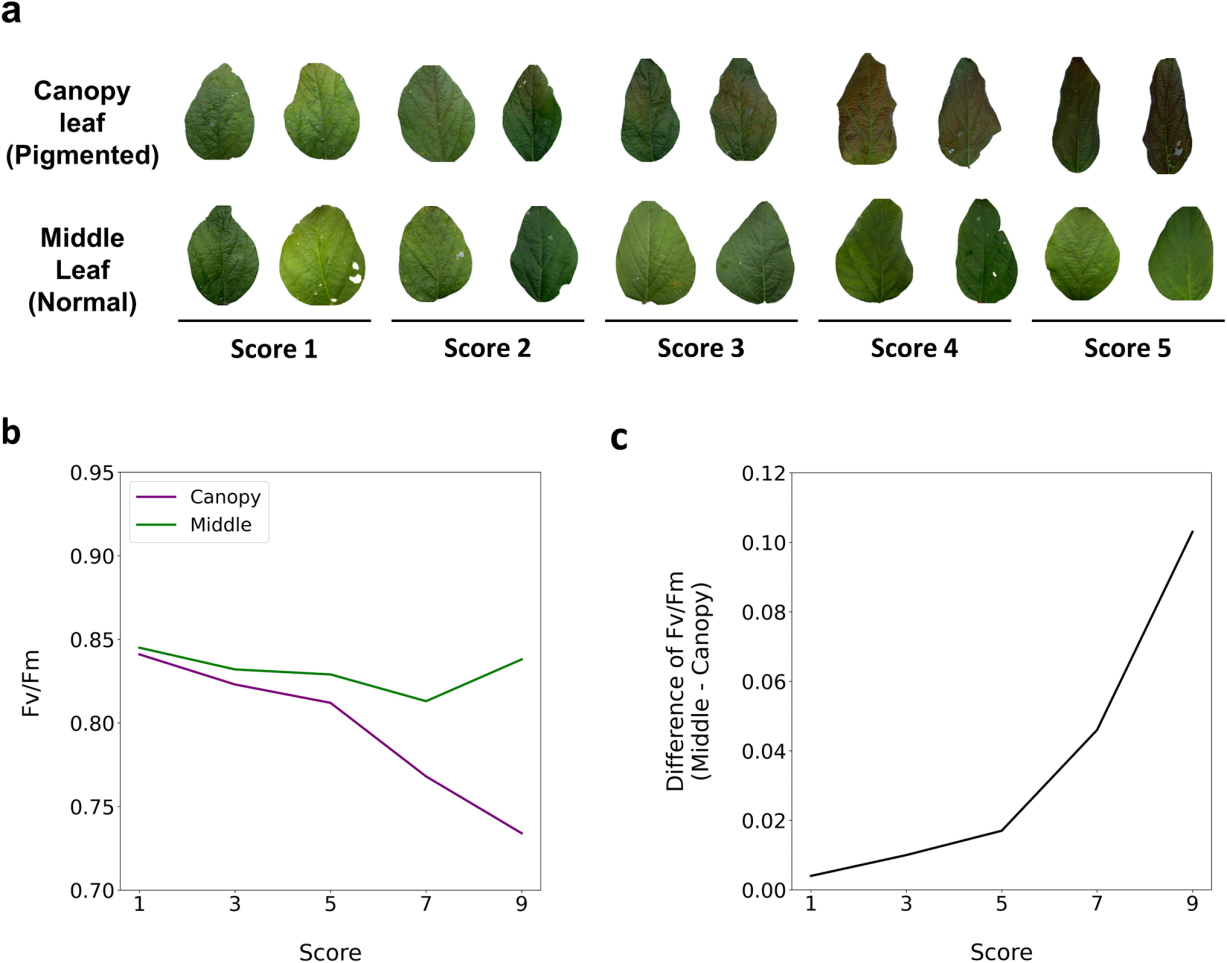
Phenotypic differences between the upper canopy and middle leaves of the selected recombinant inbred lines (RILs) with representing each pigmentation score. **a** Images of leaves from each RIL. **b** Maximum quantum yield (*F*_v_/*F*_m_) measured in both the pigmented and normal leaves of each RIL. **c** Mean differences in *F*_v_/*F*_m_ between the canopy and middle leaves in the selected RILs. The gap increased gradually as the visual score increased

The color of canopy leaves was distinguishable from that of the middle leaves in the selected RILs, with a score of 7 or 9 (Fig. 3a). The *F*_v_/*F*_m_ of PSII decreased in the canopy leaves of genotypes with a score of 9 and are referred to as sensitive genotypes (Fig. 3b). There was no significant change in the *F*_v_/*F*_m_ of PSII in the middle leaves of the sensitive genotypes (Fig. 3b). The differences in the *F*_v_/*F*_m_ values between the canopy and middle leaves increased as the pigmentation levels increased from 1 to 9 (Fig. 3c). In the genotypes with a score of 1, the changes in the measured *F*_v_/*F*_m_ values of the canopy and middle leaves were not significant, and the differences between the two positions were consistent without a dramatic increase or decrease.

### Hyperspectral imaging detected differential reflectance at 520–550 nm wavelength

**Fig. 4:**
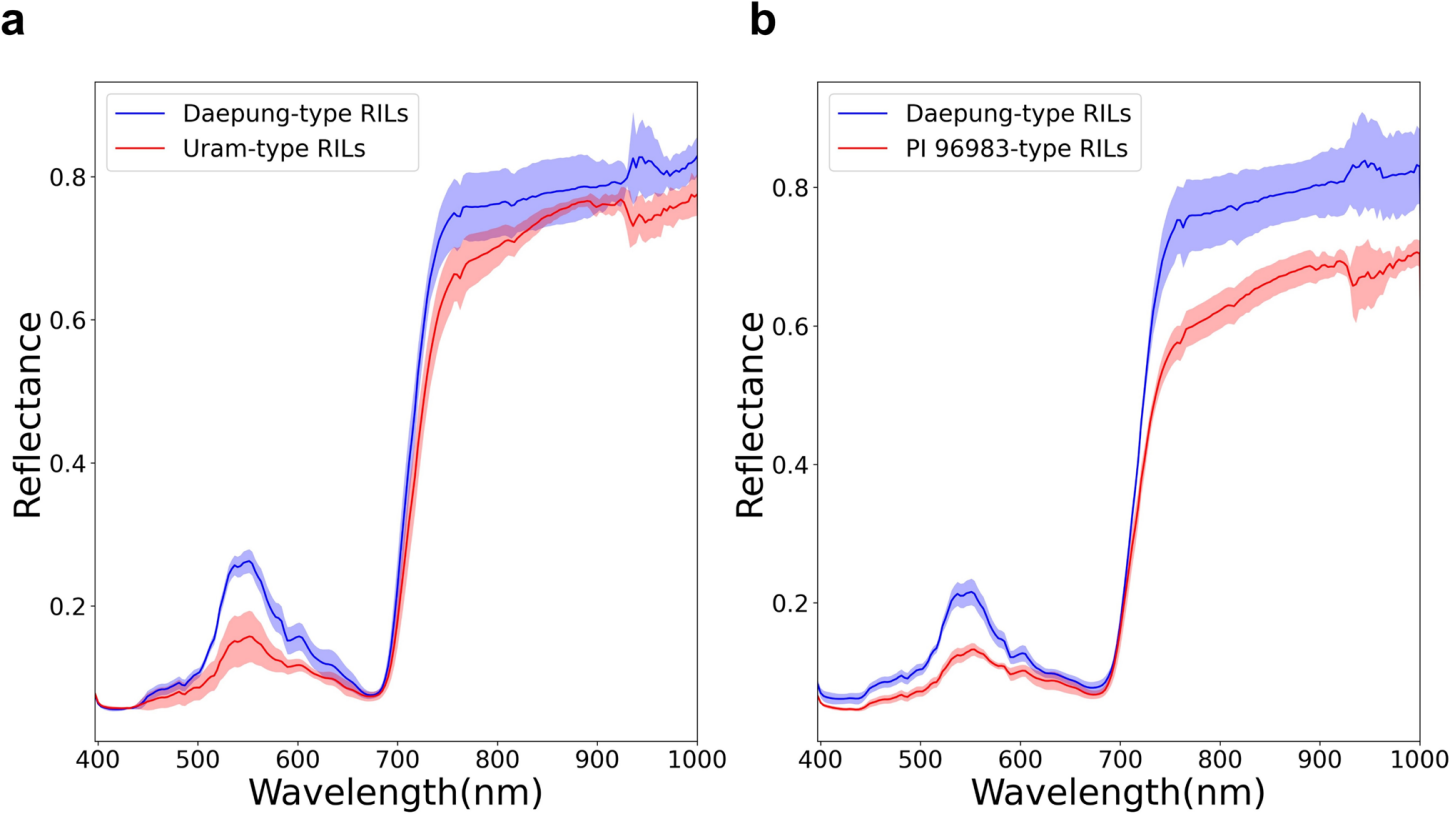
Hyperspectral reflectance of selected recombinant inbred lines (RILs) showing the discriminative pattern in the 520–550 nm range (circled area). **a** Daepung-type (score 1) versus Uram-type RILs (score 9). **b** Daepung-type (score 1) versus PI 96983-type RILs (score 7)

A comparison of spectral reflectance from the canopy leaves of the selected RILs identified wavelengths showing differential reflectance between sensitive (Uram or PI 96983-type) and insensitive (Daepung-type) genotypes (Fig. 4). The reflectance was observed at wavelengths ranging from 397 to 1000 nm from the selected RILs of the two populations, consisting of visible (397–750 nm) and infrared (750–1000 nm) regions of the spectrum that are generally assessed to characterize leaf pigmentation. The reflectance measurements exhibited a large-magnitude, distinctive pattern at 520–550 nm that was discriminative for pigmented and non-pigmented canopy leaves. The reflectance in the infrared spectrum of sensitive RILs from both populations was lower than that of the Daepung-type RILs.

### Cellular structure changes in highly pigmented canopy leaves

**Fig. 5:**
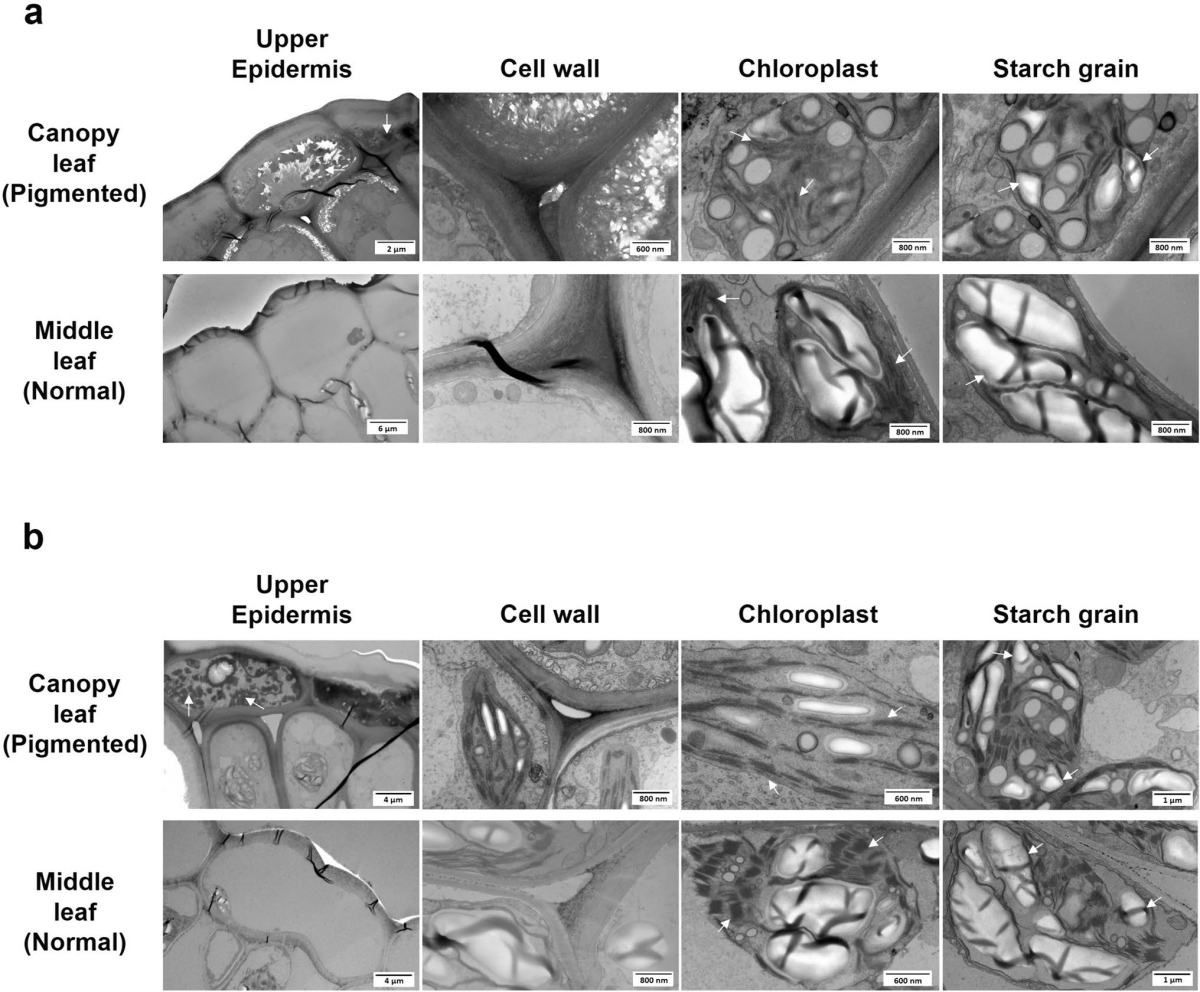
Transmission electron microscopy images highlighting the cellular differences between the pigmented canopy leaves and unpigmented middle leaves of highly sensitive recombinant inbred lines (RILs)

The cellular ultrastructure and organelles were compared between the pigmented canopy leaves and green middle leaves of one sensitive genotype from each population (10-064 and 12-002) via TEM imaging (Fig. 5). When compared to the middle leaves, increased pigments (indicated by an arrow) were found in the upper epidermal cells of the canopy leaves of both genotypes (Fig. 5). The cell wall thickness of the canopy leaves (229 ± 21 and 231 ± 32 nm, respectively, for 10-064 and 12-002) was greater than that of the middle leaves (130 ± 12 and 138 ± 19 nm, respectively) in the two genotypes (*t*-test, *P* = 0.003 and 0.029, respectively). The grana and stroma lamellae were neatly arranged in the chloroplasts of the middle leaves, whereas a thinner, disordered arrangement, along with reduced numbers of thylakoids, was observed in the canopy leaves of the two genotypes (Fig. 5). An increased number of smaller starch grains were observed in the canopy leaves, whereas swollen starch grains were observed in the middle leaves. The starch grain cover index of the canopy leaves (15.3% ± 0.4% and 15.7% ± 1.5% for 10-064 and 12-002, respectively) was significantly lower than that of the middle leaves (24.3% ± 1.2% and 38.5% ± 4.4% for 10-064 and 12-002, respectively) in the both genotypes (*t*-test, *P* = 0.008 and 0.018, respectively).

### Construction of a genetic linkage map

From a total of 169,028 SNPs, many SNPs with monomorphism, low quality, and a high portion of missing values were filtered out, resulting in 20,826 and 26,279 qualified polymorphic SNPs for NAM10 and NAM12 populations, respectively. After binning, 1177 and 1825 polymorphic SNPs, respectively, which were evenly distributed over 20 chromosomes, were retained and used to construct genetic maps of each population (Fig S1). Detailed information on the maps is summarized in Tables S3 and S4. The number of SNPs integrated in each chromosome ranged from 23 to 142, with an average of 59 and 91 in the NAM10 and NAM12 populations, respectively. The total lengths of the genetic maps were 2771 and 2946 cM, with average marker intervals of 2.4 cM in the NAM10 population and 1.6 cM in the NAM12 population. These genetic maps were validated by confirming the collinearity between the genetic position (cM) and physical position (bp), of the integrated SNPs based on the reference genome Glyma.Wm82.a2 (Fig. S2). Linear correlations were plotted in the euchromatic regions of all chromosomes in both populations (Fig. S2). Because polymorphic SNPs were missing in a few parts of two chromosomes in NAM10, an increased average distance was found between SNPs on the two chromosomes.

### Joint population analysis identified a major QTL associated with canopy leaf pigmentation changes

**Fig. 6:**
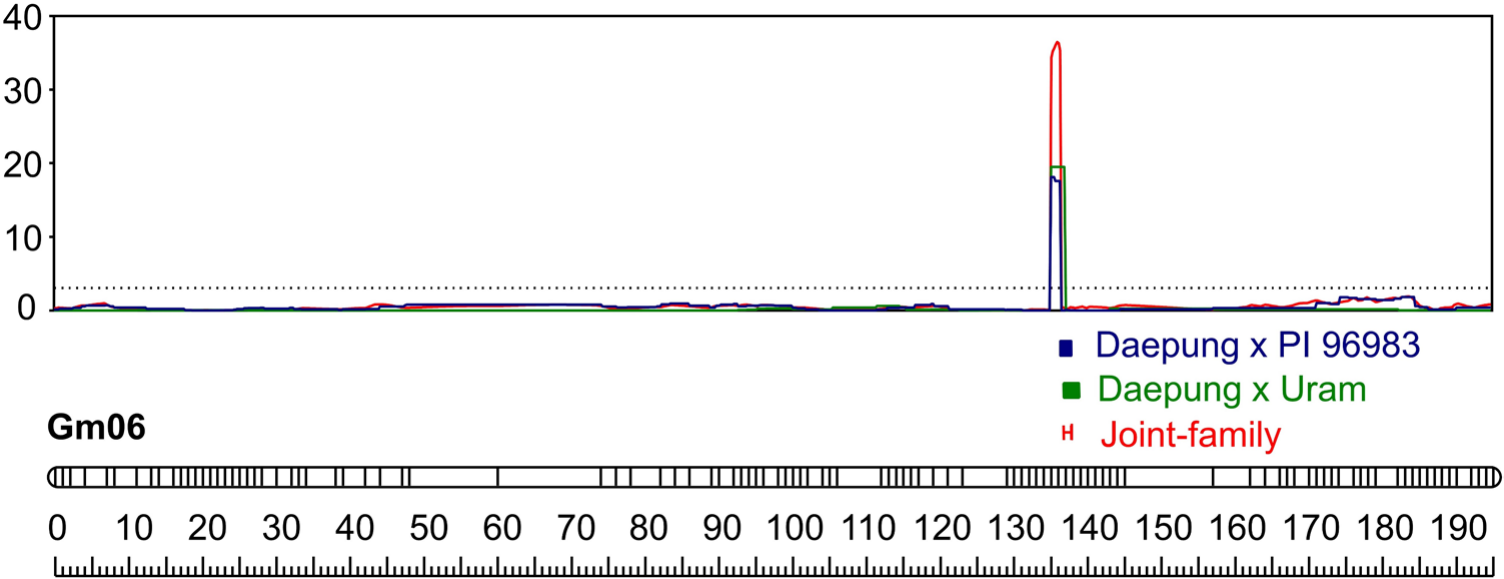
Major quantitative trait loci (QTL) associated with canopy leaf pigmentation changes identified by single and joint population QTL analyses

Using the averaged phenotypic data from across the four environmental conditions, a joint population QTL analysis was conducted on > 160 RILs from two connected RIL populations that shared a common Daepung female parent. A major QTL region between AX-90419981 and AX-90417099 at from 17.9 to 18.9 Mbp on chromosome 6 with a high LOD (i.e., ~ 36.5) was detected, which explained ~ 62.8% of the phenotypic variance (Table 3). The peak LOD was on AX-90314604 at 18.3 Mbp. The major QTL was significant for all four environmental conditions in both NAM10 and NAM12 (Table 3; Fig. 6; Fig. S3). Based on the single-population analysis of NAM10, a major QTL for CLPC was detected in the range of 17.9–19.0 Mbp on chromosome 6 that explained up to 66% of the phenotypic variance and was consistently identified in all four environments (Table S5; Fig. S3). Similarly, a major QTL between 17.9 and 18.8 Mbp on chromosome 6 was consistently identified in all environments in NAM12, explaining up to 64.4% of the phenotypic variance (Table S6; Fig. S3).

**Table 3:** Major quantitative trait locus associated with canopy leaf pigmentation changes on chromosome 6 identified by joint and individual population QTL analyses

Year	Flanking marker		Joint population		Daepung × Uram		Daepung × PI 96983	
	Left marker (bp)^a^	Right marker (bp)^a^	LOD^b^	PVE (%)^c^	LOD^b^	AE^d^	LOD^b^	AE^d^
2018	AX-90419981(17997824)	AX-90417099 (18898910)	31.2	54.3	17.1	− 2.0	14.1	− 2.4
2019	AX-90419981 (17997824)	AX-90417099 (18898910)	21.0	41.4	12.8	− 1.5	8.2	− 1.1
2020	AX-90419981 (17997824)	AX-90417099 (18898910)	26.0	51.0	15.8	− 1.8	10.2	− 1.4
2021	AX-90419981 (17997824)	AX-90417099 (18898910)	29.7	53.6	17.6	− 2.1	12.1	− 2.0
Averaged	AX-90419981 (17997824)	AX-90417099 (18898910)	36.5	62.8	21.8	− 2.0	14.6	− 1.8

^a^Physical positions (base pairs) based on the soybean reference genome Glyma.Wm82.a.2 are in parentheses

^b^Log of odds (LOD). The LOD threshold (i.e., 3.1) was determined using a 1000-permutation test

^c^Phenotypic variance (%) explained (PVE) by quantitative trait locus (QTL) analysis

^d^Additive effect

### A frameshift mutation resulted in a nonsense codon in candidate gene Glyma.06G202300

**Fig. 7:**
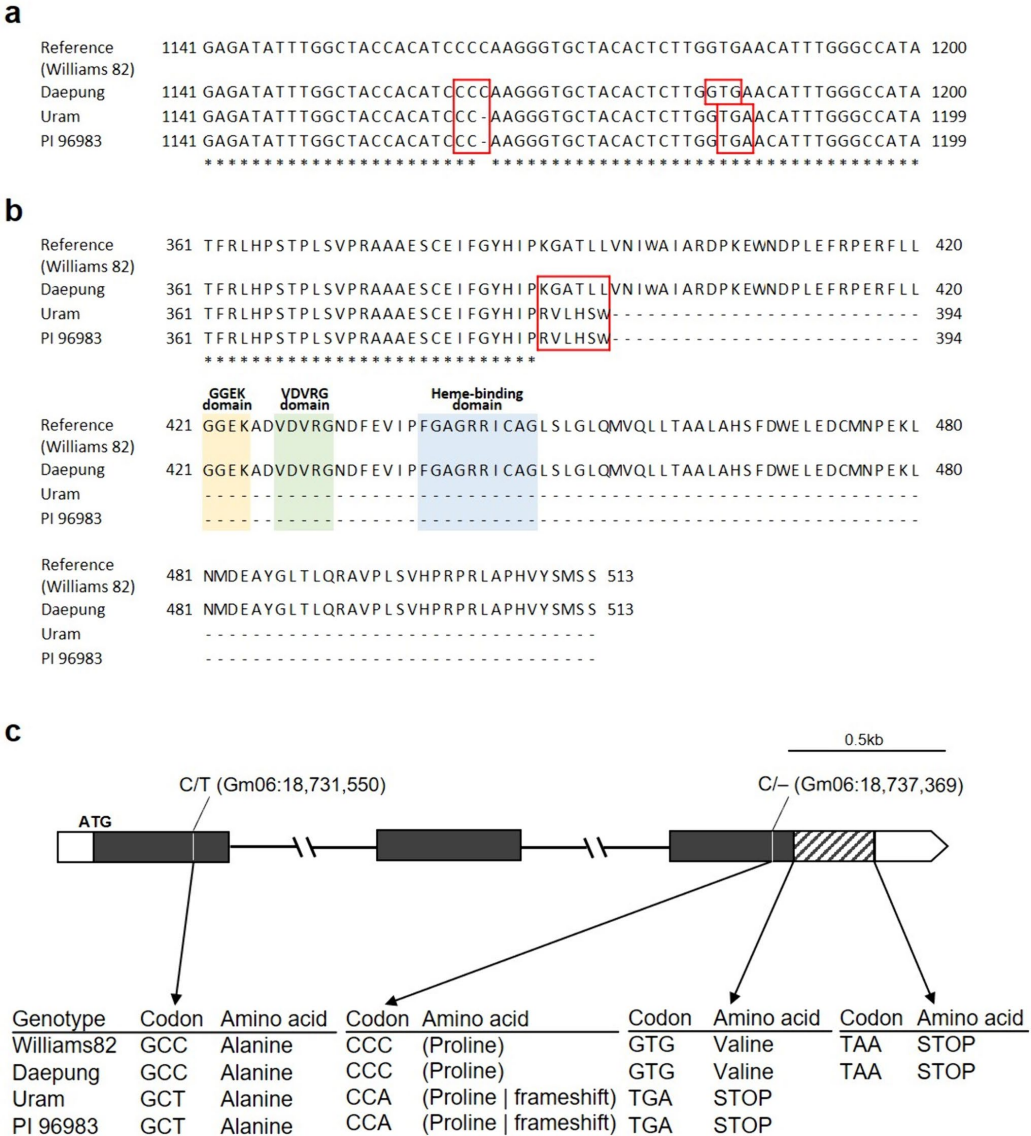
Nucleotide (**a**) and amino acid (**b**) sequence variations in exon 3 of the F3′H gene (Glyma.06G202300) among the three parental genotypes. **c** The structure of Glyma.06G202300 and the positions of mutations between the parental lines. The black boxes indicate exons, while the white boxes indicate untranslated regions. Unlike Daepung, Uram and PI 96983 have a frameshift mutation that causes the premature termination codon (TGA), producing a truncated protein with 394 amino acids (color figure online)

To identify candidate genes for the leaf pigmentation trait, we investigated a major QTL on chromosome 6 ranging from 17.9 to 18.9 Mbp. According to the reference genome sequence Glyma.Wm82.a2.v1 (www.soybase.org), a total of 46 gene models were annotated in the identified interval on chromosome 6, including those encoding exocyst complex proteins (Glyma.06G200300, Glyma.06G200400 Glyma.06G200500, Glyma.06G200600, and Glyma.06G201000), DNA-directed RNA polymerase (Glyma.06G198400), helicases (Glyma.06G198700, Glyma.06G199300, and Glyma.06G201700), ATP-dependent caseinolytic protease (Glyma.06G202200), basic helix-loop-helix/leucine zipper transcription factor (Glyma.06G199600), and flavonoid 3′-hydroxylase (Glyma.06G202300) (Table S7). Among these potential candidate genes, Glyma.06G202300, which encodes flavonoid 3′-hydroxylase (F3′H), is involved in the anthocyanin biosynthesis pathway. Sanger sequencing was performed on the genomic DNA of the parental lines to identify variations in the Glyma.06G202300. Multiple alignment analyses revealed one SNP and single-base deletions in the exons of the Glyma.06G202300. One SNP was the result of a T–to–C substitution at position 18,731,550 bp, and a single-nucleotide C was deleted at 18,737,369 bp, based on the reference genome Glyma.Wm82.a2 (Fig. 7). The mutation at 18,731,550 bp was silent; however, a frameshift mutation occurred because of the single-base deletion at 18,737,369 bp, which resulted in a premature stop codon in Uram and PI 96983 that produced a truncated protein, lacking the important conserved amino acid sequences of F3′H, such as GGEK, VDVRG, and the heme-binding domain (Toda et al. 2002) (Fig. 7).

### Differential expression of Glyma.06G202300 between pigmented and non-pigmented leaves

**Fig. 8:**
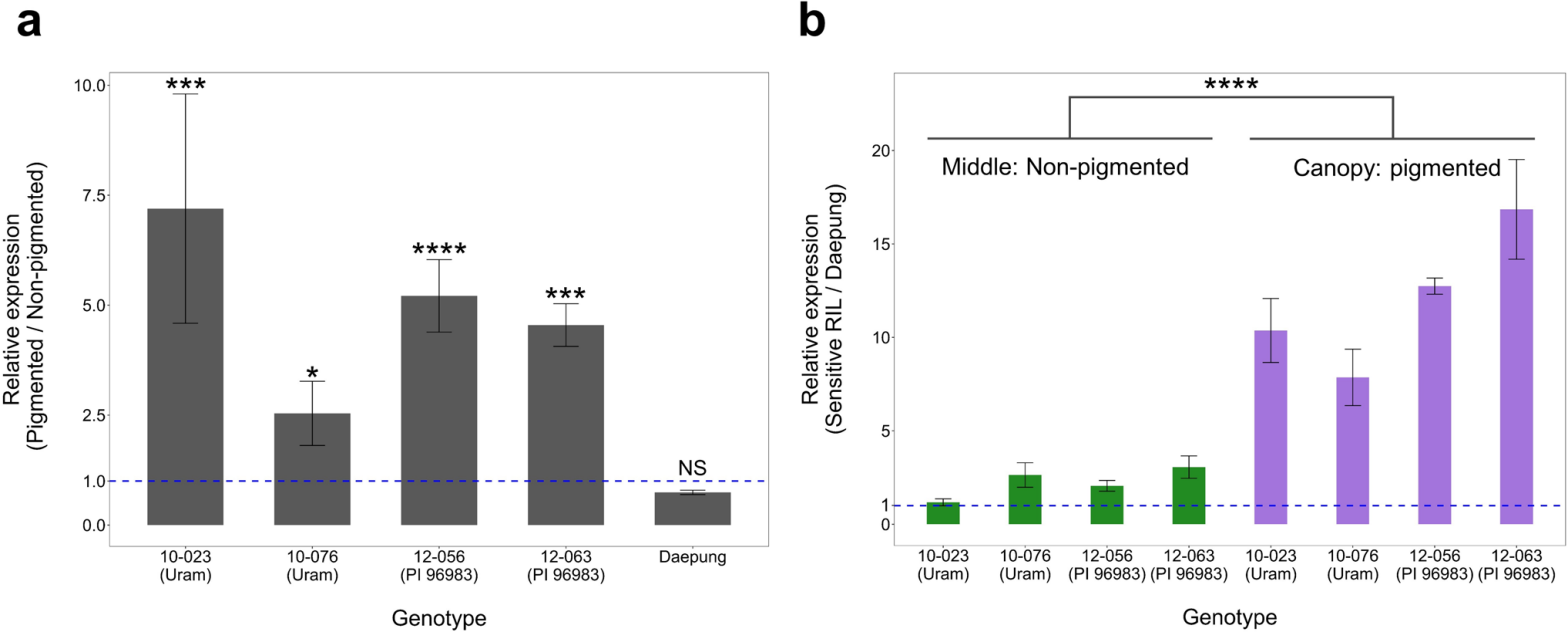
**a** Expression of Glyma.06G202300 in pigmented canopy leaves relative to that of normal middle leaves in sensitive RILs and insensitive Daepung. **b** The differential expression of Glyma.06G202300 between sensitive genotypes and Daepung in the pigmented canopy and non-pigmented middle leaves, respectively. The allele for the major QTL was indicated in the parenthesis below each RIL identification, and the hatched line denotes the value representing equal gene expression. Asterisks indicate statistical significance levels: *, *P* < 0.05; **, *P* < 0.01; ***, *P* < 0.005; ****, *P* < 0.001; *NS* non-significant

Relative expression levels of Glyma.06G202300 were characterized in two-way comparisons. First, the relative expression of canopy vs. middle leaves was calculated in the selected sensitive individuals and the Daepung parent (Fig. 8a). The gene expression levels of Glyma.06G202300 were differential by leaf position and genotypes, with 2.5- to 7.2-fold higher expression (*P* = 0.03–0.0004) in the pigmented canopy leaves than the non-pigmented middle leaves of the sensitive genotypes, while Daepung canopy leaves showed an 0.8-fold change (*P* = 0.260) of Glyma.06G202300 gene expression in middle leaves (Fig. 8a). Secondly, the relative expression levels of the sensitive RILs relative to those of the Daepung cultivar were quantified and then compared between pigmented canopy leaves and non-pigmented middle leaves. The increased expression seen in the pigmented canopy leaves of sensitive individuals was significantly higher than that seen in the middle leaves (Fig. 8b). These findings indicate that Glyma.06G202300 exhibited a specific increase in canopy leaves of sensitive RILs, suggesting its potential involvement in the CLPC.

## Discussion

The present study investigated a newly emerging leaf pigmentation change of the upper canopy during the reproductive stages of soybean development (Fig. 1). This phenomenon is presumed to be an adaptive response caused by undefined abiotic stress. To the best of our knowledge, the present study constitutes the first report of CLPC in soybean under natural conditions. Though the phenotype of UV-treated leaves is similar to that of CLPC, the major QTL identified in the response to UV (Yoon et al. 2019) was distinct from the genomic region identified in the present study. No other similar symptoms have been found in soybean. To characterize this interesting phenotype, highly pigmented canopy leaves were compared to normal (green) leaves using multiple approaches: chlorophyll fluorescence, hyperspectral reflectance, and cellular imaging. Phenotypic assays during four consecutive years also evaluated the yearly variations in pigmentation in the canopy leaves of two RIL populations under natural field conditions, and subsequently, genetic mapping identified a major QTL associated with the pigmentation change.

Sensor-based digitalized phenotyping technology, including chlorophyll fluorescence and hyperspectral reflectance, was applied to further compare the pigmented canopy leaves with normal leaves positioned in the middle of the main stem of the selected RILs. The parameter* F*_v_/*F*_m_ is a widely used indicator of the capacity of plants to convert light energy into chemical energy, and its sensitivity to various stresses allows it to be commonly used to assess the health of the photosynthetic system (Baker 2008; Maxwell and Johnson 2000). Decreased *F*_v_/*F*_m_ values can indicate damage to PSII or its inhibition by biotic factors, abiotic factors, or both (Cen et al. 2017; Ivanov and Bernards 2016; Mathur et al. 2023; Shomali et al. 2023). In soybeans, exposure to cold temperatures (22/14 °C, day/night) caused damage to the acceptor side of PSII reducing *F*_v_/*F*_m_ values to 0.69–0.78, regardless of the maturity groups (Mathur et al. 2023). Tomato leaves exposed to intense light (1200 μmol m^−2^ s^−1^) exhibited *F*_v_/*F*_m_ ratios below 0.77, contrasting with ratios of approximately 0.80 in those exposed to standard light conditions (300 μmol m^−2^ s^−1^) (Shomali et al. 2023). The current study observed decreased *F*_v_/*F*_m_ ratios (< 0.73) in the pigmented canopy leaves, while the middle leaves appear to be in good health, with a *F*_v_/*F*_m_ value of > 0.82. This indicates potential stress in the pigmentation-changed canopy leaves.

Spectral absorption and reflectance offer an alternative assessment method for leaf pigmentation (Gitelson et al. 2001; Yancheva et al. 2016). The accumulation of anthocyanin in stressed and senescing leaves was measured using the anthocyanin reflectance index (ARI). Leaf pigments were increased in plants under biotic and abiotic stress, resulting in the yellowing of leaves and increased ARI values (Gitelson et al. 2001). In the present study, reflectance wavelengths in the 520–550 nm range, which are known as the peak reflectance for anthocyanins, were significantly different between reddish-purple and green leaves (Fig. 4), indicating that an accumulation of certain flavonoids or anthocyanins is involved in the pigmented canopy leaves. In the present study, however, anthocyanin was not detected in either the pigmented or non-pigmented leaves of all tested samples (Fig. S4), which indicates that the pigmentation is associated with other flavonoids. Further studies are required to quantitatively assess other flavonoids or related secondary metabolites in the pigmented canopy leaves.

The cellular changes detected by the TEM images suggest that the pigmented canopy leaves are stressed by unidentified environmental factors. In addition to the pigments, differences in cell wall thickness, number of thylakoids, and starch granules were identified between the pigmented canopy and non-pigmented middle leaves within the same soybean plant (Fig. 5). Previous studies have documented cellular ultrastructure changes in response to environmental stressors, although no research has focused on novel CLPC in soybean. Chloroplasts under high-intensity light have fewer thylakoids per grana, larger starch grains, and many larger plastoglobuli than chloroplasts under normal conditions (Lichtenthaler and Burkart 1999). In grapevines, smaller-sized starch granules and plastoglobuli have been reported in chloroplasts under heat stress (Ben Salem-Fnayou et al. 2011). Additionally, in soybean plants, under light-shaded conditions, the number of chloroplasts and the thickness of the grana lamella increased, but the cell wall, chloroplasts, and starch grain size decreased compared with those under normal light (Fan et al. 2019). Our results are similar to the results in these previous studies; in which thinner and disordered thylakoids in reduced numbers and an increased number of smaller starch grains were observed in the leaves under abnormal conditions (Fig. 5). Thus, it is logical to suggest that certain abiotic stress might cause such cellular changes and may regulate the accumulation of phytochemicals in the symptomatic canopy leaves.

In field evaluations under natural conditions, the absence of replicated field plots within each environment rendered it impossible to distinguish between the effects of inter-environmental variations and intra-field variations. However, the phenotypic values of each RIL remained consistent and reliable across the four environments, spanning two geographical locations and four years, resulting in high levels of heritability (Table 1, Fig. 2). Subsequently, linkage analyses consistently detected a highly significant major QTL for the pigmentation in all four environments. This study first identified the major QTL associated with CLPC in soybean using joint population and individual population QTL analyses, in which LOD scores peaked near AX-90314604 at 18.3 Mbp. Joint population QTL analyses are known to enhance the power of QTL detection and increase recombination frequency by allowing the use of an integrated linkage map for more refined intervals in plant species (Li et al. 2011; Negeri et al. 2011). In this study, the LOD score from the joint population QTL analysis was much higher than those of the single-population QTL analyses, indicating to increase the power of QTL detection (Table 3). Additionally, the interval on chromosome 6 identified in the joint population analysis agreed well with those from the individual population analyses of NAM10 and NAM12 (Fig. 6). Several studies reported QTL near the locus detected in this study associated with leaf damage by ozone exposure (Burton et al. 2016), photoperiod insensitivity (Liu and Abe 2009), photo-thermal sensitivity (Wang et al. 2015), and canopy coverage (Xavier et al. 2017).

The candidate gene Glyma.06G202300 encodes F3′H, a well-known important enzyme in the anthocyanin biosynthesis pathway (Toda et al. 2002). The F3′H enzyme also controls the production of quercetin glycosides belonging to the flavonol-type flavonoid subclass in soybean leaves (Buttery and Buzzell 1973; Pratt 1976; Toda et al. 2011). Phenolic (anthocyanins), terpenes (carotenoids), alkaloids, and nitrogen-containing metabolites play an important role in protection of plants from biotic and abiotic stresses (Liu et al. 2023). The accumulation of secondary metabolites including anthocyanin showed the differences in different regions of same plant species because of the variation in light conditions (Zhang et al. 2021). Colored flavonoids play a pivotal role in the responses to various environmental stresses, exhibiting photoprotective effects (Hughes et al. 2005; Liang and He 2018; Moustaka et al. 2020). A recent study demonstrated that accumulation of colored flavonoids under exposure to high light stress with *Arabidopsis* showed better photoprotections as a light attenuation which was more important than antioxidative photoprotections (Zheng et al. 2021). Further studies will aim to investigate changes in phytochemicals related to CLPC, because it may provide photoprotective effects on the canopy under stress conditions caused by light intensity or quality.

Canopy structure and health are key features in plant growth and development and thus are often used to predict soybean yield in agricultural applications (Schmitz and Kandel 2021; Xavier et al. 2017). Canopy wilting, greenness, and defoliation caused by several biotic and abiotic stresses during reproductive stages affect soybean yield (Board et al. 2010; Hou et al. 2019; Thrash et al. 2021; Ye et al. 2020; Yoon et al. 2019). The current study did not aim to clarify the effect of CLPC on soybean seed quality or production yield of soybean, but it is still worth discussing its potential impacts on soybean agronomy. Several studied reported that the F3′H gene was associated with seed yield and quality of soybean (Funatsuki et al. 2005; Toda et al. 2011). Regarding seed quality, a soybean genotype with the f3′h allele showed the development of pigmentation around the hilum of soybean seed and increased seed coat cracking under chilling temperature exposure (Takahashi and Asanuma 1996). Seed yields were not significantly different between near-isogenic lines with the F3′H or f3′h allele under normal conditions but were differentially reduced by 24% and 45% under chilled conditions, respectively (Takahashi and Asanuma 1996; Takahashi et al. 2005). Additionally, increased antioxidant activities due to the production of quercetin and 3′, 4′-dihydroxylated flavonol were suggested to be associated with yield differences under chilled conditions (Buttery and Buzzell 1973; Pratt 1976; Toda et al. 2011). In the RILs of the two mapping populations, all harvested seeds presented normal phenotype and no cracked seeds were found during the four years of experiments. Trivial levels of purple-seed stain caused by the fungus *Cercospora kikuchii* were observed in only a few lines. The existence and nature of any pleiotropic effects of CLPC on seed quality and quantity will be an interesting subject to study in further experiments.

In conclusion, this study characterized physiological responses in pigmented canopy leaves, investigated changes in ultracellular structure and organelles, and identified a highly significant major QTL on chromosome 6 based on the multi-environment field tests. The TEM observation and measured physiological features, such as the *F*_v_/*F*_m_ of PSII, and hyperspectral reflectance, indicated that the pigment-changed canopy leaves were affected by undefined environmental stressors and subsequently accumulated pigments. Despite expectations for the accumulation of anthocyanins due to pigmentation, it was surprising to find that anthocyanin was not detected in 14 pigmented leaf samples. Further studies will determine which flavonoids and secondary metabolites are involved in the CLPC in soybean. This study also provides unique information because only a few have looked at leaf pigments other than chlorophyll, while many previous studies have focused on elucidating the molecular mechanisms of seed coat pigmentation in soybean. This study revealed that F3′H (Glyma.06G202300) may be an important regulator of the pigmentation change of canopy leaves in soybean. Overall, our findings provide valuable information for genomic research and may benefit marker-assisted selection in breeding programs aimed at producing soybean varieties adapted to climate change.

## Supplementary Information

Below is the link to the electronic supplementary material.

Supplementary file1 (PDF 2170 kb)

Supplementary file2 (XLSX 30 kb)

## References

[CR1] Baker NR (2008) Chlorophyll fluorescence: a probe of photosynthesis in vivo. Annu Rev Plant Biol 59:89–113 18444897 10.1146/annurev.arplant.59.032607.092759

[CR2] Ben Salem-Fnayou A, Bouamama B, Ghorbel A, Mliki A (2011) Investigations on the leaf anatomy and ultrastructure of grapevine (*Vitis vinifera*) under heat stress. Microsc Res Tech 74:756–762 21780249 10.1002/jemt.20955

[CR3] Bhat JA, Ali S, Salgotra RK, Mir ZA, Dutta S, Jadon V, Tyagi A, Mushtaq M, Jain N, Singh PK, Singh GP, Prabhu KV (2016) Genomic selection in the *Era* of next generation sequencing for complex traits in plant breeding. Front Genet 7:221 28083016 10.3389/fgene.2016.00221 PMC5186759

[CR4] Blackburn GA (1998) Spectral indices for estimating photosynthetic pigment concentrations: a test using senescent tree leaves. Int J Remote Sens 19:657–675 10.1080/014311698215919

[CR5] Board J, Kumudini S, Omielan J, Prior E, Kahlon C (2010) Yield response of soybean to partial and total defoliation during the seed-filling period. Crop Sci 50:703–712 10.2135/cropsci2009.03.0128

[CR6] Burton AL, Burkey KO, Carter TE, Orf J, Cregan PB (2016) Phenotypic variation and identification of quantitative trait loci for ozone tolerance in a Fiskeby III × Mandarin (Ottawa) soybean population. Theor Appl Genet 129:1113–1125 26920548 10.1007/s00122-016-2687-1

[CR7] Buttery BR, Buzzell RI (1973) Varietal differences in leaf flavonoids of Soybeans. Crop Sci 13:103–106 10.2135/cropsci1973.0011183X001300010033xa

[CR8] Carey CC, Strahle JT, Selinger DA, Chandler VL (2004) Mutations in the pale aleurone color1 regulatory gene of the *Zea mays* anthocyanin pathway have distinct phenotypes relative to the functionally similar TRANSPARENT TESTA GLABRA1 gene in *Arabidopsis thaliana*. Plant Cell 16:450–464 14742877 10.1105/tpc.018796 PMC341916

[CR9] Cen H, Weng H, Yao J, He M, Lv J, Hua S, Li H, He Y (2017) Chlorophyll fluorescence imaging uncovers photosynthetic fingerprint of citrus huanglongbing. Front Plant Sci. 10.3389/fpls.2017.01509 28900440 10.3389/fpls.2017.01509 PMC5581828

[CR10] Chandler VL, Radicella JP, Robbins TP, Chen J, Turks D (1989) Two regulatory genes of the maize anthocyanin pathway are homologous: isolation of B utilizing R genomic sequences. Plant Cell 1:1175–1183 2535537 10.1105/tpc.1.12.1175 PMC159853

[CR11] Chappelle EW, Kim MS, McMurtrey JE (1992) Ratio analysis of reflectance spectra (RARS): an algorithm for the remote estimation of the concentrations of chlorophyll A, chlorophyll B, and carotenoids in soybean leaves. Remote Sens Environ 39:239–247 10.1016/0034-4257(92)90089-3

[CR12] Churchill GA, Doerge RW (1994) Empirical threshold values for quantitative trait mapping. Genetics 138:963–971 7851788 10.1093/genetics/138.3.963 PMC1206241

[CR13] Cone KC, Burr FA, Burr B (1986) Molecular analysis of the maize anthocyanin regulatory locus C1. Proc Natl Acad Sci USA 83:9631–9635 3025847 10.1073/pnas.83.24.9631 PMC387194

[CR77] Djanaguiraman M, Prasad PV, Boyle DL, Schapaugh WT (2011) High-temperature stress and soybean leaves: Leaf anatomy and photosynthesis. Crop Sci 51(5):2125–2131 10.2135/cropsci2010.10.0571

[CR14] Dhondt S, Wuyts N, Inzé D (2013) Cell to whole-plant phenotyping: the best is yet to come. Trends Plant Sci 18:428–439 23706697 10.1016/j.tplants.2013.04.008

[CR15] Duddu HSN, Johnson EN, Willenborg CJ, Shirtliffe SJ (2019) High-throughput UAV image-based method is more precise than manual rating of herbicide tolerance. Plant Phenomics 2019:6036453 33313532 10.34133/2019/6036453 PMC7706330

[CR16] Fan Y, Chen J, Wang Z, Tan T, Li S, Li J, Wang B, Zhang J, Cheng Y, Wu X, Yang W, Yang F (2019) Soybean (Glycine max L. Merr.) seedlings response to shading: leaf structure, photosynthesis and proteomic analysis. BMC Plant Biol 19:34 30665369 10.1186/s12870-019-1633-1 PMC6341755

[CR17] Funatsuki H, Kawaguchi K, Matsuba S, Sato Y, Ishimoto M (2005) Mapping of QTL associated with chilling tolerance during reproductive growth in soybean. Theor Appl Genet 111:851–861 16059730 10.1007/s00122-005-0007-2

[CR76] Gao M, Liu Y, Ma X, Shuai Q, Gai J, Li Y (2017) Evaluation of reference genes for normalization of gene expression using quantitative RT-PCR under aluminum, cadmium, and heat stresses in soybean. PLoS ONE 12(1):e0168965. 10.1371/journal.pone.0168965 28046130 10.1371/journal.pone.0168965 PMC5207429

[CR18] Gamon JA, Surfus JS (1999) Assessing leaf pigment content and activity with a reflectometer. New Phytol 143:105–117 10.1046/j.1469-8137.1999.00424.x

[CR19] Gitelson A, Merzlyak MN (1994) Spectral reflectance changes associated with autumn senescence of *Aesculus hippocastanum* L. and *Acer platanoides* L. leaves. Spectral features and relation to chlorophyll estimation. J Plant Physiol 143:286–292 10.1016/S0176-1617(11)81633-0

[CR20] Gitelson AA, Merzlyak MN, Chivkunova OB (2001) Optical properties and nondestructive estimation of anthocyanin content in plant leaves. Photochem Photobiol 74:38–45 11460535 10.1562/0031-8655(2001)074<0038:OPANEO>2.0.CO;2

[CR21] Haak DC, Fukao T, Grene R, Hua Z, Ivanov R, Perrella G, Li S (2017) Multilevel regulation of abiotic stress responses in plants. Front Plant Sci. 10.3389/fpls.2017.01564s 29033955 10.3389/fpls.2017.01564s PMC5627039

[CR22] Holton TA, Cornish EC (1995) Genetics and biochemistry of anthocyanin biosynthesis. Plant Cell 7:1071–1083 12242398 10.2307/3870058 PMC160913

[CR23] Hou M, Tian F, Zhang T, Huang M (2019) Evaluation of canopy temperature depression, transpiration, and canopy greenness in relation to yield of soybean at reproductive stage based on remote sensing imagery. Agric Water Manag 222:182–192 10.1016/j.agwat.2019.06.005

[CR24] Hughes NM, Neufeld HS, Burkey KO (2005) Functional role of anthocyanins in high-light winter leaves of the evergreen herb *Galax urceolata*. New Phytol 168:575–587 16313641 10.1111/j.1469-8137.2005.01546.x

[CR25] Ivanov DA, Bernards MA (2016) Chlorophyll fluorescence imaging as a tool to monitor the progress of a root pathogen in a perennial plant. Planta 243:263–279 26537710 10.1007/s00425-015-2427-9

[CR78] Jian B, Liu B, Bi Y, Hou W, Wu C, Han T (2008) Validation of internal control for gene expression study in soybean by quantitative real-time PCR. BMC Mol Biol 9:1–14 18573215 10.1186/1471-2199-9-59 PMC2443375

[CR79] Khaleghi A, Naderi R, Brunetti C, Maserti BE, Salami SA, Babalar M (2019) Morphological, physiochemical and antioxidant responses of* Maclura pomifera *to drought stress. Sci Rep 9(1):19250 31848429 10.1038/s41598-019-55889-y PMC6917715

[CR26] Kiihl RAS, Hartwig EE (1979) Inheritance of reaction to soybean mosaic virus in Soybeans. Crop Sci 19:372–375 10.2135/cropsci1979.0011183X001900030024x

[CR27] Kim J-T, Yi G, Chung I-M, Son B-Y, Bae H-H, Go YS, Ha JY, Baek S-B, Kim S-L (2020) Timing and pattern of anthocyanin accumulation during grain filling in purple waxy corn (*Zea mays* L.) suggest optimal harvest dates. ACS Omega 5:15702–15708 32637845 10.1021/acsomega.0c02099 PMC7331206

[CR28] Kim KH (2018) Genome-wide association analysis of flowering time genes with nested association mapping (NAM) population in soybean. Master Thesis, DanKook University, Cheonan, Korea

[CR29] Kitajima M, Butler WL (1975) Quenching of chlorophyll fluorescence and primary photochemistry in chloroplasts by dibromothymoquinone. Biochim Biophys Acta 376:105–115 1125215 10.1016/0005-2728(75)90209-1

[CR30] Ko JM, Han WY, Kim HT, Lee YH, Choi MS, Lee BW, Shin SU, Seo JH, Oh KW, Yun HT, Jeon MG, Choi KH, Shin JH, Lee EJ, Yang S, Oh IS (2016) Soybean cultivar for soy-paste, ‘Uram’ with mechanization harvesting, large seed, disease resistance and high yield. Korean J Breed Sci 48:301–306 10.9787/KJBS.2016.48.3.301

[CR31] Krishnan HB, Kim WS, Oehrle NW, Smith JR, Gillman JD (2020) Effect of heat stress on seed protein composition and ultrastructure of protein storage vacuoles in the cotyledonary parenchyma cells of soybean genotypes that are either tolerant or sensitive to elevated temperatures. Int J Mol Sci 21:4775 32635665 10.3390/ijms21134775 PMC7370294

[CR32] Lee YG, Jeong N, Kim JH, Lee K, Kim KH, Pirani A, Ha BK, Kang ST, Park BS, Moon JK, Kim N, Jeong SC (2015) Development, validation and genetic analysis of a large soybean SNP genotyping array. Plant J 81:625–636 25641104 10.1111/tpj.12755

[CR33] Lepiniec L, Debeaujon I, Routaboul J-M, Baudry A, Pourcel L, Nesi N, Caboche M (2006) Genetics and biochemistry of seed flavonoids. Annu Rev Plant Biol 57:405–430 16669768 10.1146/annurev.arplant.57.032905.105252

[CR34] Li H, Ye G, Wang J (2007) A modified algorithm for the improvement of composite interval mapping. Genetics 175:361–374 17110476 10.1534/genetics.106.066811 PMC1775001

[CR35] Li H, Bradbury P, Ersoz E, Buckler ES, Wang J (2011) Joint QTL linkage mapping for multiple-cross mating design sharing one common parent. PLoS ONE 6:e17573 21423655 10.1371/journal.pone.0017573 PMC3057965

[CR36] Liang J, He J (2018) Protective role of anthocyanins in plants under low nitrogen stress. Biochem Biophys Res Commun 498:946–953 29548824 10.1016/j.bbrc.2018.03.087

[CR37] Lichtenthaler H, Burkart S (1999) Photosynthesis and high light stress. Bulg J Plant Physiol 25:3–16

[CR80] Lippmann R, Babben S, Menger A, Delker C, Quint M (2019) Development of wild and cultivated plants under global warming conditions. Curr Biol 29(24):R1326–R1338 31846685 10.1016/j.cub.2019.10.016

[CR38] Liu B, Abe J (2009) QTL Mapping for photoperiod insensitivity of a Japanese soybean landrace Sakamotowase. J Hered 101:251–256 19959597 10.1093/jhered/esp113

[CR81] Liu Y, Cai Y, Li Y, Zhang X, Shi N, Zhao J, Yang H (2022) Dynamic changes in the transcriptome landscape of *Arabidopsis thaliana* in response to cold stress. Front Plant Sci 13:983460 36110360 10.3389/fpls.2022.983460 PMC9468617

[CR39] Liu Y, Singh SK, Pattanaik S, Wang H, Yuan L (2023) Light regulation of the biosynthesis of phenolics, terpenoids, and alkaloids in plants. Commun Biol 6:1055 37853112 10.1038/s42003-023-05435-4 PMC10584869

[CR40] Lowe A, Harrison N, French AP (2017) Hyperspectral image analysis techniques for the detection and classification of the early onset of plant disease and stress. Plant Methods 13:80 29051772 10.1186/s13007-017-0233-z PMC5634902

[CR41] Mathur S, Seo B, Jajoo A, Reddy KR, Reddy VR (2023) Chlorophyll fluorescence is a potential indicator to measure photochemical efficiency in early to late soybean maturity groups under changing day lengths and temperatures. Front Plant Sci. 10.3389/fpls.2023.1228464 37936935 10.3389/fpls.2023.1228464 PMC10627226

[CR42] Maxwell K, Johnson GN (2000) Chlorophyll fluorescence—a practical guide. J Exp Bot 51:659–668 10938857 10.1093/jexbot/51.345.659

[CR43] Meng L, Li H, Zhang L, Wang J (2015) QTL IciMapping: integrated software for genetic linkage map construction and quantitative trait locus mapping in biparental populations. Crop J 3:269–283 10.1016/j.cj.2015.01.001

[CR44] Merzlyak MN, Gitelson AA, Chivkunova OB, Rakitin VY (1999) Non-destructive optical detection of pigment changes during leaf senescence and fruit ripening. Physiol Plant 106:135–141 10.1034/j.1399-3054.1999.106119.x

[CR45] Moustaka J, Tanou G, Giannakoula A, Adamakis I-DS, Panteris E, Eleftheriou EP, Moustakas M (2020) Anthocyanin accumulation in *poinsettia* leaves and its functional role in photo-oxidative stress. Environ Exp Bot 175:104065 10.1016/j.envexpbot.2020.104065

[CR46] Negeri AT, Coles ND, Holland JB, Balint-Kurti PJ (2011) Mapping QTL controlling southern leaf blight resistance by joint analysis of three related recombinant inbred line populations. Crop Sci 51:1571–1579 10.2135/cropsci2010.12.0672

[CR47] Nwokolo E (1996) Soybean (*Glycine max* L. Merr.). In: Nwokolo E, Smartt J (eds) Food and feed from legumes and oilseeds. Springer, Boston, pp 90–102

[CR48] O’Callaghan FE, Braga RA, Neilson R, MacFarlane SA, Dupuy LX (2018) New live screening of plant-nematode interactions in the rhizosphere. Sci Rep 8:1440 29362410 10.1038/s41598-017-18797-7 PMC5780396

[CR49] Park K-Y, Moon J-K, Yun H-T, Lee Y-H, Kim S-L, Ryu Y-H, Kim Y-H, Ku J-H, Roh J-H, Lee E-S, Ha K-S, Kim I-j, Son C-K, Kim S-K, Kim S-D, Moon H-P (2005) A new soybean cultivar for fermented soyfood and tofu with high yield, “Daepung.” Korean J Breed Sci 37:111–112

[CR50] Pratt DE (1976) Role of flavones and related compounds in retarding lipid—oxidative flavor changes in foods. Phenolic, sulfur, and nitrogen compounds in food flavors. Am Chem Soc. 10.1021/bk-1976-0026.ch001 10.1021/bk-1976-0026.ch001

[CR51] Ramakrishna A, Ravishankar GA (2011) Influence of abiotic stress signals on secondary metabolites in plants. Plant Signal Behav 6:1720–1731 22041989 10.4161/psb.6.11.17613 PMC3329344

[CR52] Reddy V (1998) Cloning and characterization of the rice homologue of the maize *C1* anthocyanin regulatory gene. Plant Mol Biol 36:497–498 10.1023/A:1017106913186

[CR84] Ruelland E, Vaultier MN, Zachowski A, Hurry V (2009) Cold signalling and cold acclimation in plants. Adv Bot Res 49:35–150 10.1016/S0065-2296(08)00602-2

[CR82] Schmittgen TD, Livak KJ (2008) Analyzing real-time PCR data by the comparative CT method. Nat Protoc 3(6):1101–1108 18546601 10.1038/nprot.2008.73

[CR53] Schmitz PK, Kandel HJ (2021) Using canopy measurements to predict soybean seed yield. Remote Sensing 13:3260 10.3390/rs13163260

[CR54] Shah A, Smith DL (2020) Flavonoids in agriculture: chemistry and roles in, biotic and abiotic stress responses, and microbial associations. Agronomy 10:1209 10.3390/agronomy10081209

[CR83] Sharma M, Kumar P, Verma V, Sharma R, Bhargava B, Irfan M (2022) Understanding plant stress memory response for abiotic stress resilience: molecular insights and prospects. Plant Physiol Biochem 179:10–24 35305363 10.1016/j.plaphy.2022.03.004

[CR55] Shomali A, Aliniaeifard S, Bakhtiarizadeh MR, Lotfi M, Mohammadian M, Vafaei Sadi MS, Rastogi A (2023) Artificial neural network (ANN)-based algorithms for high light stress phenotyping of tomato genotypes using chlorophyll fluorescence features. Plant Physiol Biochem 201:107893 37459804 10.1016/j.plaphy.2023.107893

[CR56] Singh AK, Singh A, Sarkar S, Ganapathysubramanian B, Schapaugh W, Miguez FE, Carley CN, Carroll ME, Chiozza MV, Chiteri KO, Falk KG, Jones SE, Jubery TZ, Mirnezami SV, Nagasubramanian K, Parmley KA, Rairdin AM, Shook JM, Van der Laan L, Young TJ, Zhang J (2021) High-throughput phenotyping in soybean. In: Zhou J, Nguyen HT (eds) High-throughput crop phenotyping. Springer, Cham, pp 129–163

[CR57] Song H, Yoon S-R, Dang Y-M, Yang J-S, Hwang IM, Ha J-H (2022) Nondestructive classification of soft rot disease in napa cabbage using hyperspectral imaging analysis. Sci Rep 12:14707 36038711 10.1038/s41598-022-19169-6 PMC9424267

[CR58] Takahashi R, Asanuma S (1996) Association of *T* gene with chilling tolerance in soybean. Crop Sci 36:559–562 10.2135/cropsci1996.0011183X003600030004x

[CR59] Takahashi R, Benitez ER, Funatsuki H, Ohnishi S (2005) Soybean maturity and pubescence color genes improve chilling tolerance. Crop Sci 45:1387–1393 10.2135/cropsci2004.0386

[CR60] Tao H, Xu S, Tian Y, Li Z, Ge Y, Zhang J, Wang Y, Zhou G, Deng X, Zhang Z, Ding Y, Jiang D, Guo Q, Jin S (2022) Proximal and remote sensing in plant phenomics: 20 years of progress, challenges, and perspectives. Plant Commun 3:100344 35655429 10.1016/j.xplc.2022.100344 PMC9700174

[CR61] Thrash BC, Catchot AL Jr, Gore J, Cook D, Musser FR, Irby T, Krutz J (2021) Effects of soybean plant population on yield loss from defoliation. J Econ Entomol 114:702–709 33503250 10.1093/jee/toaa279

[CR62] Toda K, Yang D, Yamanaka N, Watanabe S, Harada K, Takahashi R (2002) A single-base deletion in soybean flavonoid 3’-hydroxylase gene is associated with gray pubescence color. Plant Mol Biol 50:187–196 12175012 10.1023/A:1016087221334

[CR63] Toda K, Takahashi R, Iwashina T, Hajika M (2011) Difference in chilling-induced flavonoid profiles, antioxidant activity and chilling tolerance between soybean near-isogenic lines for the pubescence color gene. J Plant Res 124:173–182 20428921 10.1007/s10265-010-0345-2

[CR85] Untergasser A, Cutcutache I, Koressaar T, Ye J, Faircloth BC, Remm M, Rozen SG (2012) Primer3–new capabilities and interfaces. Nucleic Acids Res 40(15):e115–e115 22730293 10.1093/nar/gks596 PMC3424584

[CR64] Voorrips RE (2002) MapChart: Software for the graphical presentation of linkage maps and QTLs. J Hered 93:77–78 12011185 10.1093/jhered/93.1.77

[CR65] Wang Y, Cheng L, Leng J, Wu C, Shao G, Hou W, Han T (2015) Genetic analysis and quantitative trait locus identification of the reproductive to vegetative growth period ratio in soybean (*Glycine max* (L.) Merr.). Euphytica 201:275–284 10.1007/s10681-014-1209-y

[CR66] Wang L, Liu F, Hao X, Wang W, Xing G, Luo J, Zhou G, He J, Gai J (2021) Identification of the QTL-allele system underlying two high-throughput physiological traits in the Chinese soybean germplasm population. Front Genet. 10.3389/fgene.2021.600444 33719333 10.3389/fgene.2021.600444 PMC7947801

[CR67] Xavier A, Hall B, Hearst AA, Cherkauer KA, Rainey KM (2017) Genetic architecture of phenomic-enabled canopy coverage in *Glycine max*. Genetics 206:1081–1089 28363978 10.1534/genetics.116.198713 PMC5499164

[CR68] Xiao Q, Bai X, Zhang C, He Y (2022) Advanced high-throughput plant phenotyping techniques for genome-wide association studies: a review. J Adv Res 35:215–230 35003802 10.1016/j.jare.2021.05.002 PMC8721248

[CR69] Yancheva S, Lidiya G, Kostova M, Halkoglu P, Dimitrova M, Naimov S (2016) Plant pigments content as a marker for herbicide abiotic stress in Corn (*Zea mays* L.). Emir J Food Agric. 10.9755/ejfa.2016-02-135 10.9755/ejfa.2016-02-135

[CR70] Yang Y, Zheng G, Han L, Dagang W, Yang X, Yuan Y, Huang S, Zhi H (2013) Genetic analysis and mapping of genes for resistance to multiple strains of soybean mosaic virus in a single resistant soybean accession PI 96983. Theor Appl Genet 126:1783–1791 23580088 10.1007/s00122-013-2092-y

[CR71] Ye H, Song L, Schapaugh WT, Ali ML, Sinclair TR, Riar MK, Mutava RN, Li Y, Vuong T, Valliyodan B, Pizolato Neto A, Klepadlo M, Song Q, Shannon JG, Chen P, Nguyen HT (2020) The importance of slow canopy wilting in drought tolerance in soybean. J Exp Bot 71:642–652 30980084 10.1093/jxb/erz150 PMC6946001

[CR72] Yoon MY, Kim MY, Ha J, Lee T, Kim KD, Lee S-H (2019) QTL analysis of resistance to high-intensity UV-B irradiation in Soybean (*Glycine max* [L.] Merr.). Int J Mol Sci 20:3287 31277435 10.3390/ijms20133287 PMC6651677

[CR73] Zhang S, Zhang L, Zou H, Qiu L, Zheng Y, Yang D, Wang Y (2021) Effects of light on secondary metabolite biosynthesis in medicinal plants. Front Plant Sci 12:781236 34956277 10.3389/fpls.2021.781236 PMC8702564

[CR74] Zheng X-T, Yu Z-C, Tang J-W, Cai M-L, Chen Y-L, Yang C-W, Chow WS, Peng C-L (2021) The major photoprotective role of anthocyanins in leaves of *Arabidopsis thaliana* under long-term high light treatment: Antioxidant or light attenuator? Photosynth Res 149:25–40 32462454 10.1007/s11120-020-00761-8

[CR86] Zhao J, Lu Z, Wang L, Jin B (2020) Plant responses to heat stress: physiology, transcription, noncoding RNAs, and epigenetics. Int J Mol Sci 22(1):117 33374376 10.3390/ijms22010117 PMC7795586

[CR75] Zhou Y, Chen J, Ma J, Han X, Chen B, Li G, Xiong Z, Huang F (2022) Early warning and diagnostic visualization of *Sclerotinia* infected tomato based on hyperspectral imaging. Sci Rep 12:21140 36477460 10.1038/s41598-022-23326-2 PMC9729219

