# Supplementary material for: Exploration and genetic analyses of canopy leaf pigmentation changes in soybean (Glycine max L.): unveiling a novel phenotype
Source: Theor Appl Genet. 2024 Aug 13;137(9):202. doi: 10.1007/s00122-024-04693-y (PMC11319514; doi:10.1007/s00122-024-04693-y)
Supplement: Supplementary file 1 — Supplementary file1 (PDF 2170 kb) [file 122_2024_4693_MOESM1_ESM.pdf]

Supplementary Figures

Discovery and genetic analysis of a novel phenotype in soybean (*Glycine max* L.): Canopy leaf pigmentation

Hee Jin You[1,†], Hyun Jo[2,†], Jeongsun Lee[1], Seongha Kwon[1], Ji-Min Kim[3], Sung-Taeg Kang[3], Luong Ngoc Ha[1], Sungwoo Lee[1,*]

[1] Department of Crop Science, College of Agriculture and Life Sciences, Chungnam National University, Daejeon 34134, South Korea

[2] Department of Applied Biosciences, College of Agriculture and Life Sciences, Kyungpook National University, Daegu 41566, South Korea

[3] Department of Crop Science and Biotechnology, College of Bioresource Science, Dankook University, Cheonan, Chungnam 31116, South Korea

[†] These authors equally contributed to this work.

* Corresponding author: Sungwoo Lee

Tel) +82-42-821-5727, Fax) +82-42-822-2631

E-mail) : sungwoolee@cnu.ac.kr

ORCID) https://orcid.org/0000-0003-3564-236

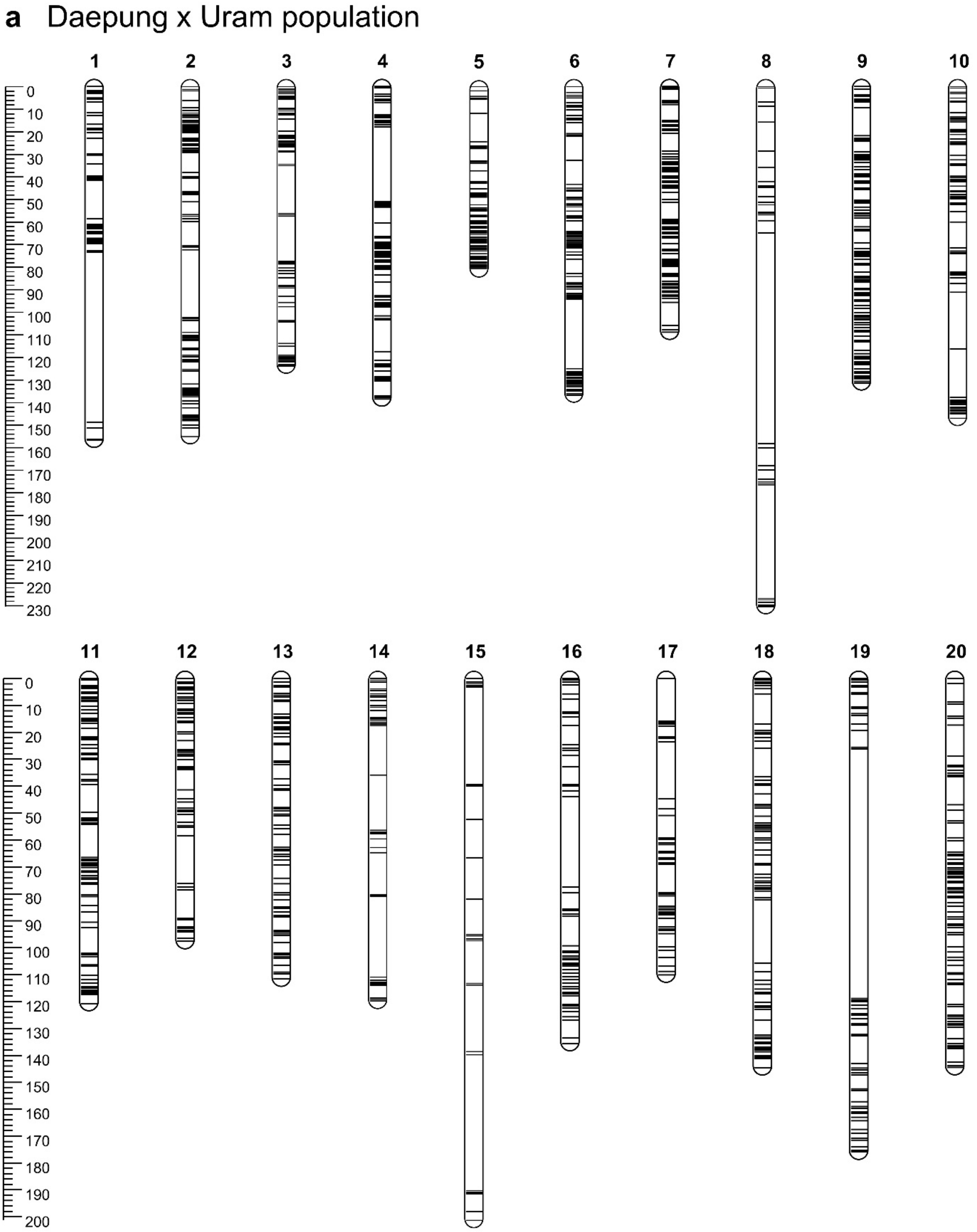

Fig. S1 Distribution of integrated single nucleotide polymorphisms (SNPs). **a** Genetic maps of the Daepung × Uram population (NAM 10). **b** Genetic map of the Daepung × PI 96983 population (NAM 12).

b Daepung x PI 96983 population

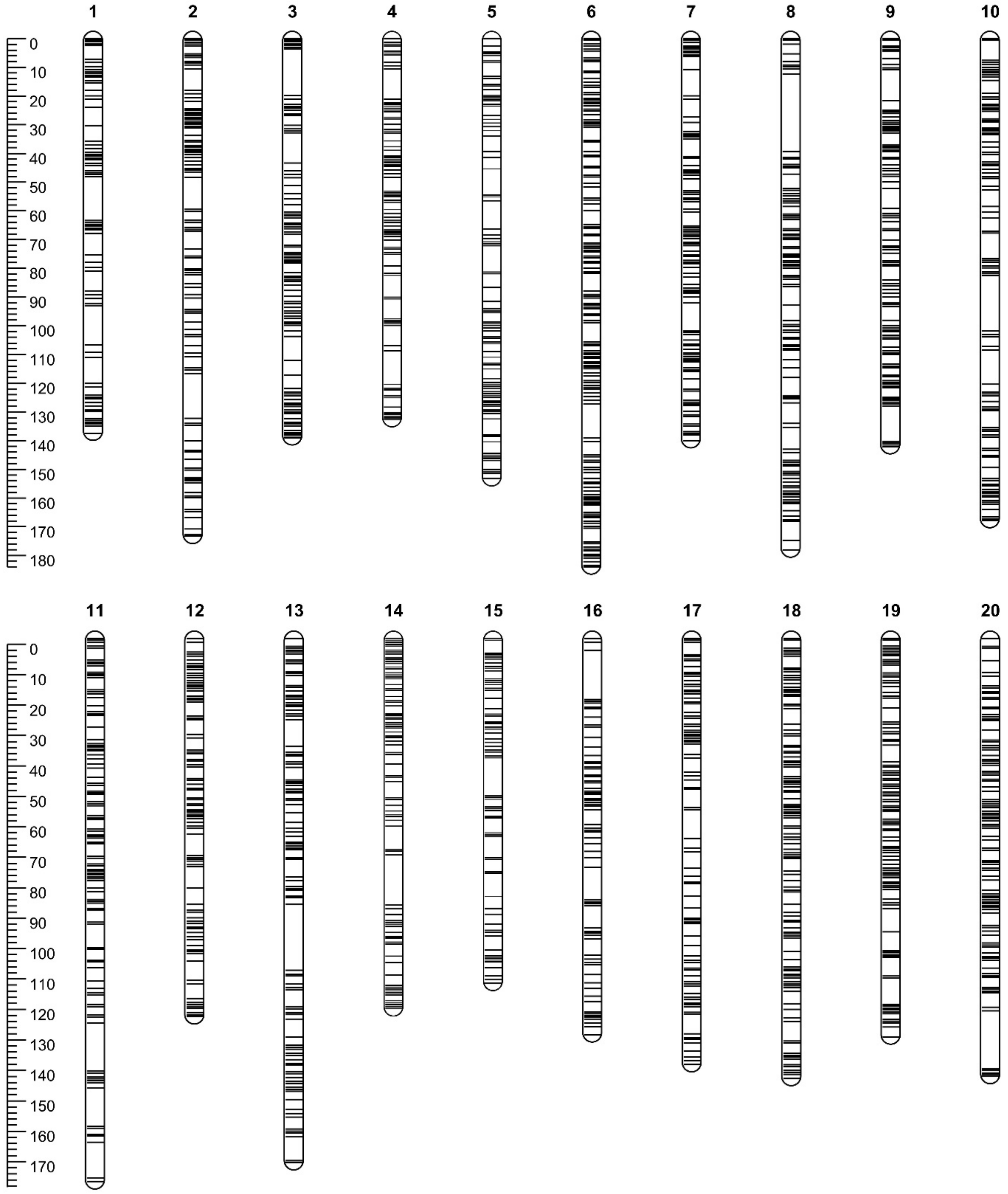

Fig. S1 Continued

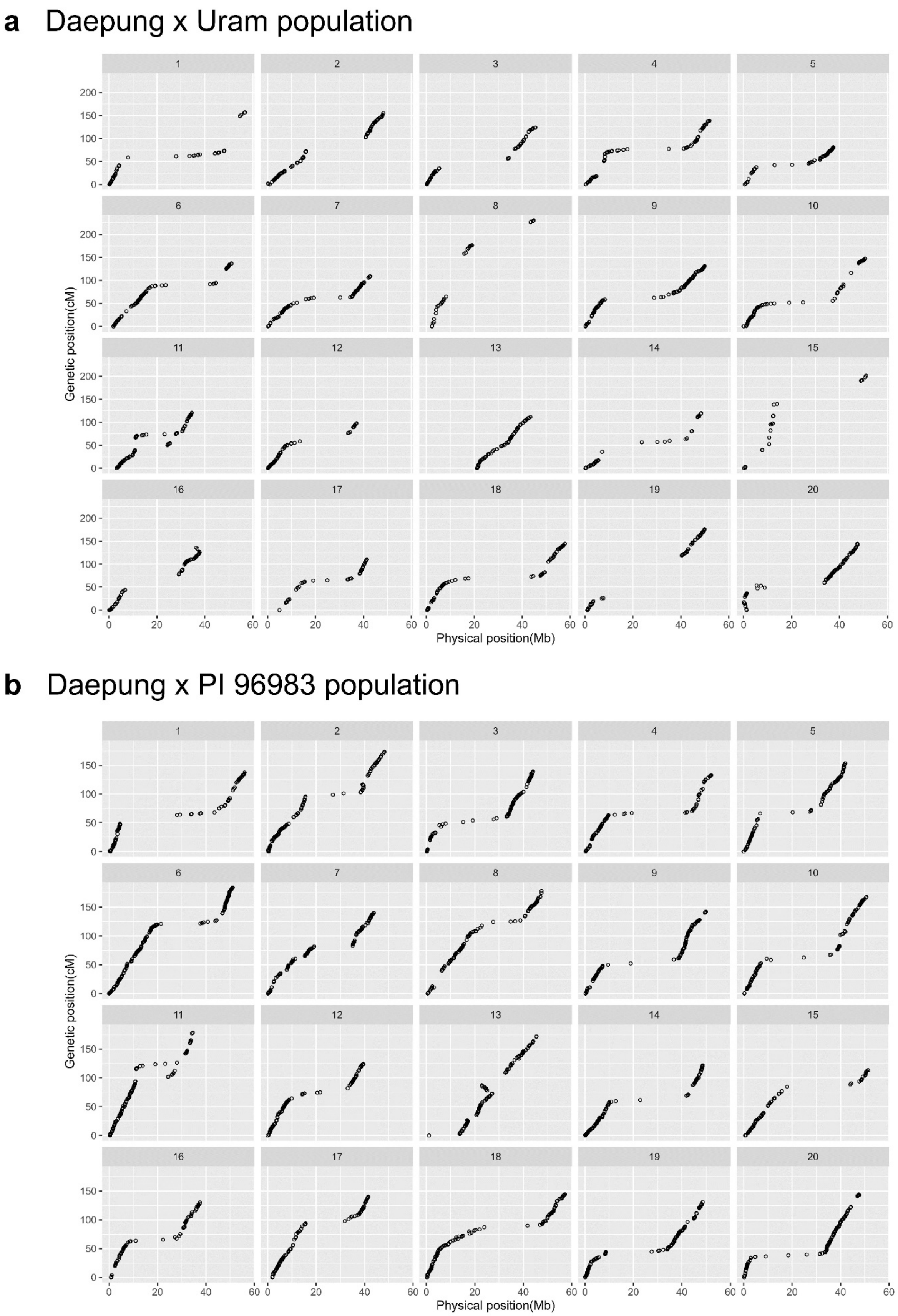

Fig. S2 Correlation between the genetic (cM) and physical (Mb) positions of the single nucleotide polymorphisms (SNPs) integrated in the genetic maps. **a** Daepung × Uram population (NAM 10). **b** Daepung × PI 96983 population (NAM 12).

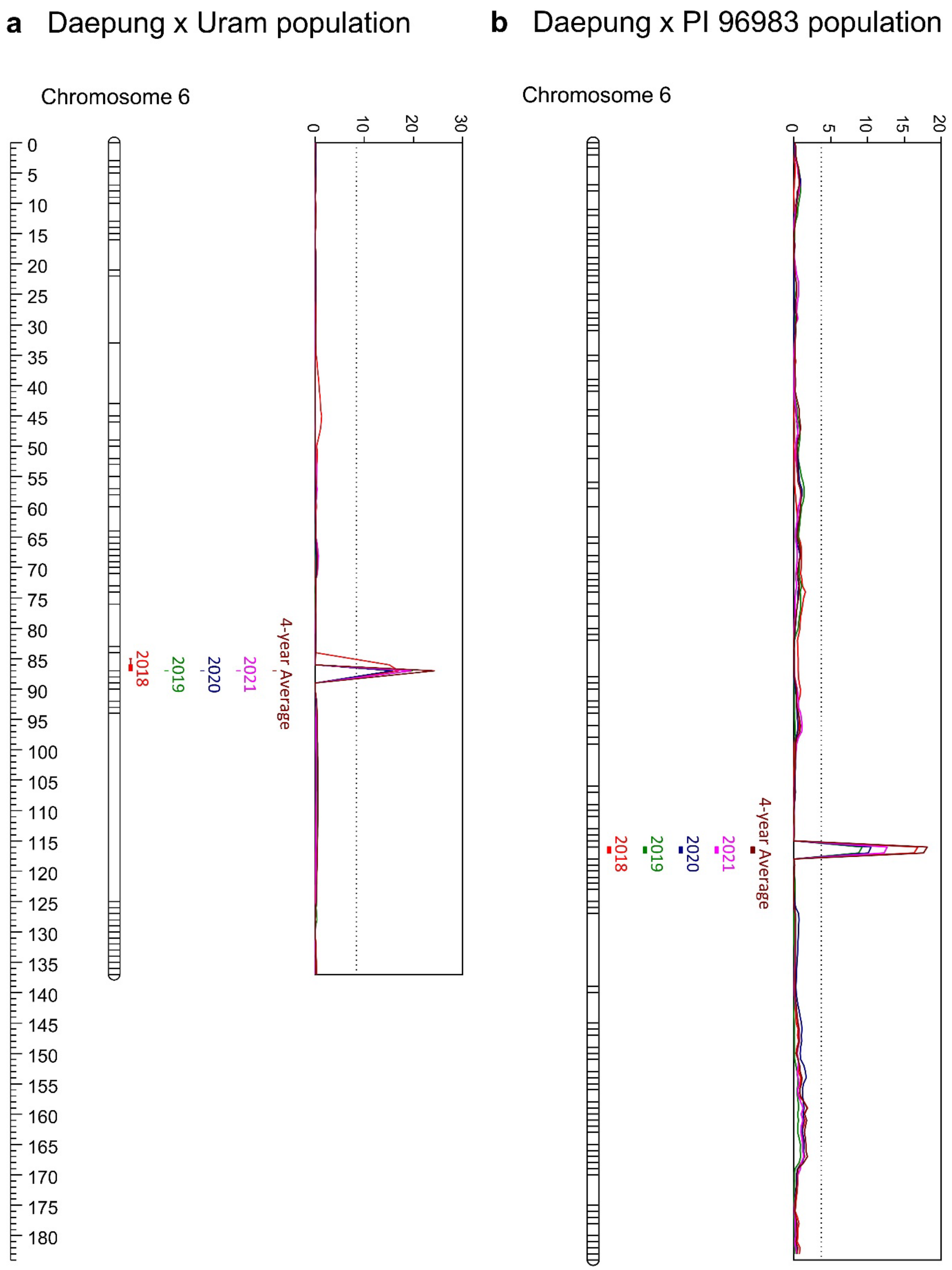

Fig. S3 Major quantitative trait locus associated with canopy leaf pigmentation for 4 years. (a) The Daepung × Uram population (NAM 10). (b) The Daepung × PI 96983 population (NAM 12).

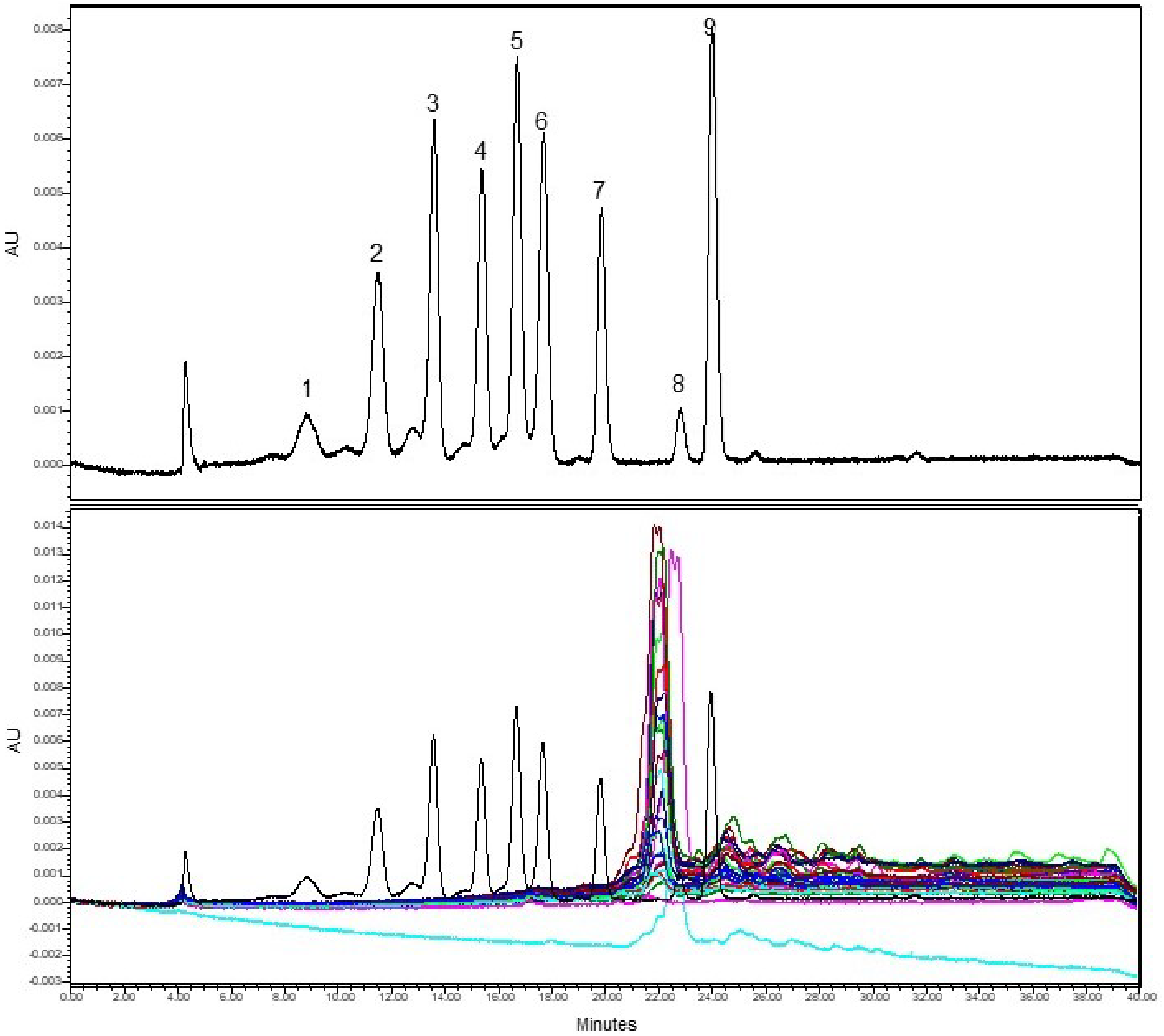

Fig. S4 The absence of anthocyanins was confirmed in highly pigmented (score 9) canopy leaves of sensitive recombinant inbred lines and Daepung through ultrahigh-performance liquid chromatography. The upper panel displays peaks of the standard mix, while the lower panel showed peaks of 28 samples alongside the standard mix. AU, absorbance units, 1: cyanin chloride, 2: pelagonin chloride, 3: kuromanin chloride (C3G), 4: callistephin chloride, 5: peonidin-3-O-glucoside chloride, 6: oenin chloride, 7: cyanidin chloride, 8: pelargonidin chloride, 9: peonidin chloride.
